# Uganda’s “EID Systems Strengthening” model produces significant gains in testing, linkage, and retention of HIV-exposed and infected infants: An impact evaluation

**DOI:** 10.1371/journal.pone.0246546

**Published:** 2021-02-04

**Authors:** Charles Kiyaga, Vijay Narayan, Ian McConnell, Peter Elyanu, Linda Nabitaka Kisaakye, Eleanor Joseph, Adeodata Kekitiinwa, Jeff Grosz

**Affiliations:** 1 Ministry of Health AIDS Control Programme, Kampala, Uganda; 2 Clinton Health Access Initiative, Kampala, Uganda; 3 Baylor College of Medicine, Kampala, Uganda; University of North Carolina at Chapel Hill, UNITED STATES

## Abstract

**Introduction:**

A review of Uganda’s HIV Early Infant Diagnosis (EID) program in 2010 revealed poor retention outcomes for HIV-exposed infants (HEI) after testing. The review informed development of the ‘EID Systems Strengthening’ model: a set of integrated initiatives at health facilities to improve testing, retention, and clinical care of HIV-exposed and infected infants. The program model was piloted at several facilities and later scaled countrywide. This mixed-methods study evaluates the program’s impact and assesses its implementation.

**Methods:**

We conducted a retrospective cohort study at 12 health facilities in Uganda, comprising all HEI tested by DNA PCR from June 2011 to May 2014 (n = 707). Cohort data were collected manually at the health facilities and analyzed. To assess impact, retention outcomes were statistically compared to the baseline study’s cohort outcomes. We conducted a cross-sectional qualitative assessment of program implementation through 1) structured clinic observation and 2) key informant interviews with health workers, district officials, NGO technical managers, and EID trainers (n = 51).

**Results:**

The evaluation cohort comprised 707 HEI (67 HIV+). The baseline study cohort contained 1268 HEI (244 HIV+). Among infants testing HIV+, retention in care at an ART clinic increased from 23% (57/244) to 66% (44/67) (p < .0001). Initiation of HIV+ infants on ART increased from 36% (27/75) to 92% (46/50) (p < .0001). HEI receiving 1st PCR results increased from 57% (718/1268) to 73% (518/707) (p < .0001). Among breastfeeding HEI with negative 1st PCR, 55% (192/352) received a confirmatory PCR test, a substantial increase from baseline period. Testing coverage improved significantly: HIV+ pregnant women who brought their infants for testing after birth increased from 18% (67/367) to 52% (175/334) (p < .0001). HEI were tested younger: mean age at DBS test decreased from 6.96 to 4.21 months (p < .0001). Clinical care for HEI was provided more consistently. Implementation fidelity was strong for most program components. The strongest contributory interventions were establishment of ‘EID Care Points’, integration of clinical care, longitudinal patient tracking, and regular health worker mentorship. Gaps included limited follow up of lost infants, inconsistent buy-in/ownership of health facility management, and challenges sustaining health worker motivation.

**Discussion:**

Uganda’s ‘EID Systems Strengthening’ model has produced significant gains in testing and retention of HEI and HIV+ infants, yet the country still faces major challenges. The 3 core concepts of Uganda’s model are applicable to any country: establish a central service point for HEI, equip it to provide high-quality care and tracking, and develop systems to link HEI to the service point. Uganda’s experience has shown the importance of intensively targeting systemic bottlenecks to HEI retention at facility level, a necessary complement to deploying rapidly scalable technologies and other higher-level initiatives.

## Introduction

Despite tremendous progress over the past decade, 150,000 children were newly infected with HIV in 2019, with 126,000 of them in sub-Saharan Africa [1]. In Uganda, over 90,000 HIV-exposed infants (HEI) are born yearly and 5,000 new children are infected with HIV [2]. To survive, HIV-positive (HIV+) infants must be diagnosed and started early on anti-retroviral therapy (ART) [3, 4]. Disease progression is rapid: if infected infants are not started on ART, 35% will likely die by their first birthday and over 50% by age two [5–7].

**Fig 1:**
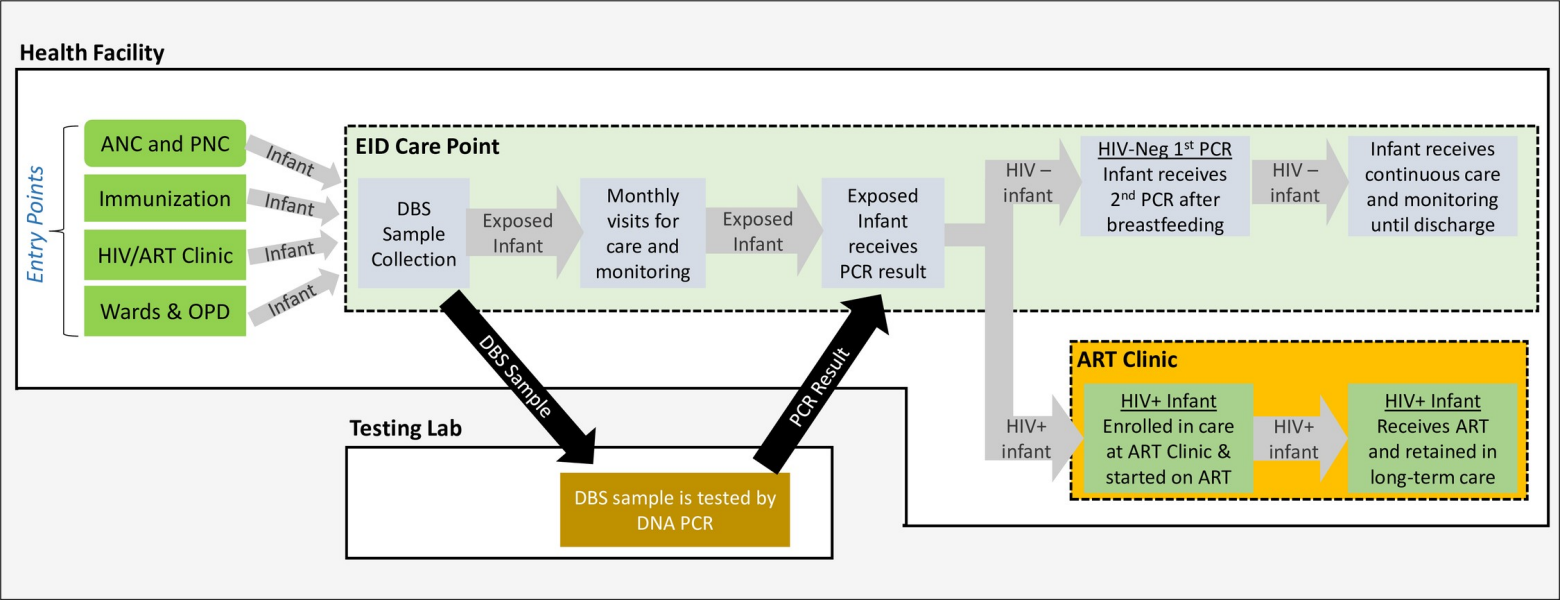
Uganda’s EID cascade. Diagram showing key steps in the HIV Early Infant Diagnosis process in Uganda. Each step represents a potential loss point for HIV-exposed and/or HIV-positive infants. ANC = Antenatal Care Clinic, PNC = Postnatal Care Clinic, OPD = Outpatient Department, DBS = Dried Blood Spot, PCR = Polymerase Chain Reaction.

Early Infant Diagnosis (EID) is a complex, multi-step process (Fig 1). In Uganda, Dried Blood Spot (DBS) samples are obtained from HEI at health facilities from 6 weeks of age. The DBS samples are transported to a specialized reference lab and tested by DNA Polymerase Chain Reaction (PCR) [8]. The PCR results are then sent back to the health facility and given to the HEI’s caregiver. If negative, a 2^nd^ confirmatory PCR is done after cessation of breastfeeding [9].

**Fig 2:**
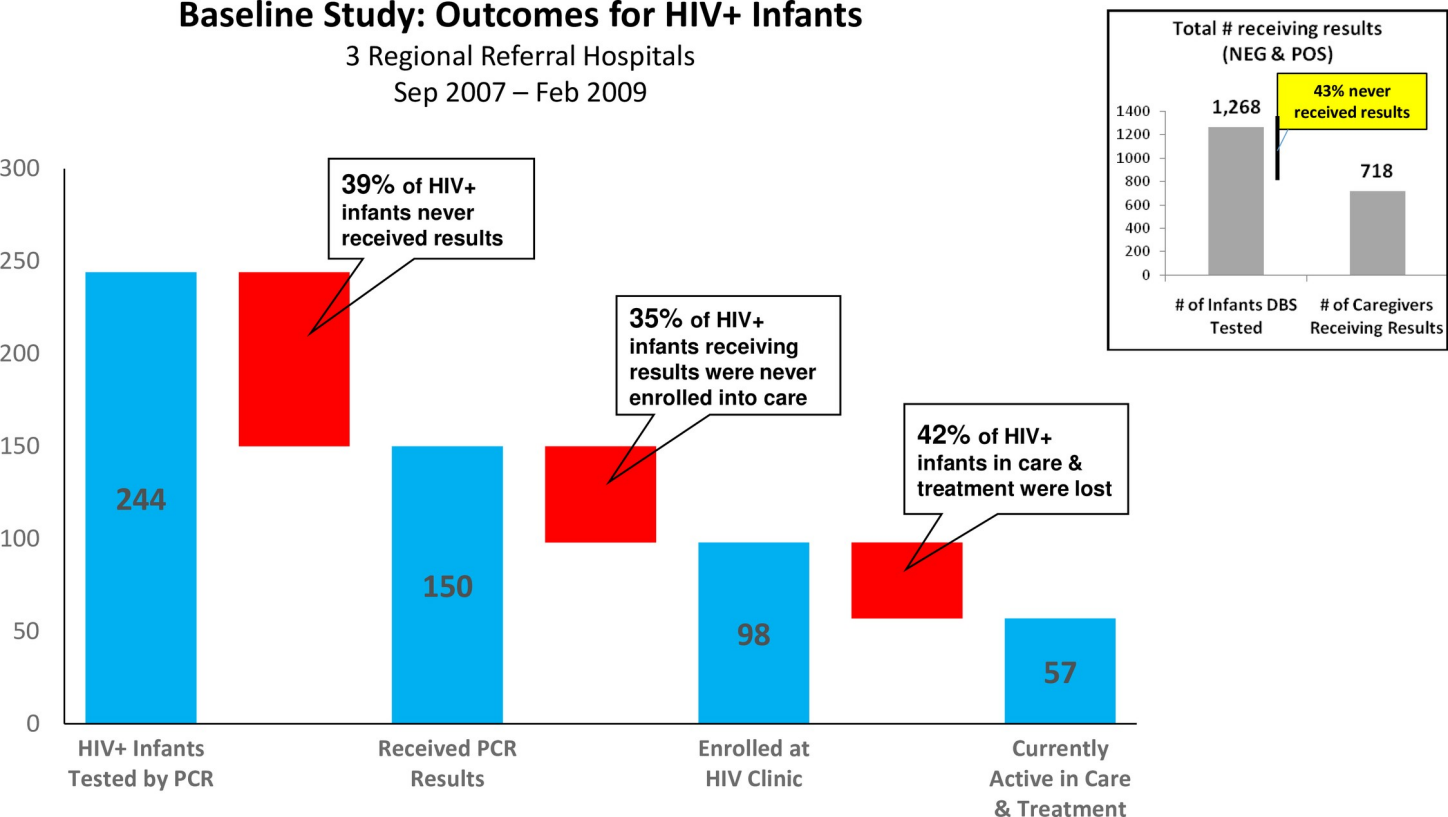
Baseline study: Outcomes for HIV+ infants. Graph showing the retention outcomes for HIV-exposed infants testing HIV-positive (n = 244) in the 2010 pre-intervention study. The red bars represent the number of infants lost to-follow up at each step in the EID cascade. The box in the top-right corner shows the percent of all tested HIV-exposed infants (n = 1268)—both negative and positive—who received their results.

Established by Uganda’s Ministry of Health (MOH) in 2007, the National EID Program rapidly scaled up testing of HEI. However, little was known about outcomes of HEI after testing. In 2010, the MOH and Clinton Health Access Initiative (CHAI) conducted a program review at 7 health facilities: a retrospective cohort analysis of tested HEI at 3 hospitals, and qualitative assessment at all 7 facilities [10]. Here we briefly highlight several key findings reported from that study, which serve as the baseline measures for our pre/post intervention comparison. Out of 1,268 HEI identified and tested at the 3 hospitals, only 57% received their PCR test results [10]. On average, HEI were tested late (M = 6.96 months, N = 1,268). Many HEI weren’t tested at all: out of 367 HIV+ pregnant women at one hospital, only 18% of their infants received a DBS test after birth. Ultimately, of the 244 infants testing HIV+ at the 3 hospitals, only 23% (57/244) were still active in care (Fig 2) [10].

The qualitative component of the 2010 study diagnosed bottlenecks driving loss of HEI at each point in the EID cascade [10]. The study found that EID services were fragmented across several clinics in the health facility, with none of them equipped for HEI care and tracking. The flow of caregivers, samples, and results within the facility was ad hoc and ineffective, contributing to HEI loss. Health workers (HWs) lacked data tools to track HEI through the EID process and identify those who defaulted [10]. Lack of intra-facility referral mechanisms hindered linkage of HEI from entry point clinics to the DBS testing point, and linkage of HIV+ infants to ART services. Clinical care was largely absent, preventing early identification of opportunistic infections. Poor counseling of HEI caregivers and inadequate HW knowledge about EID were major gaps contributing to HEI attrition [10].

**Fig 3:**
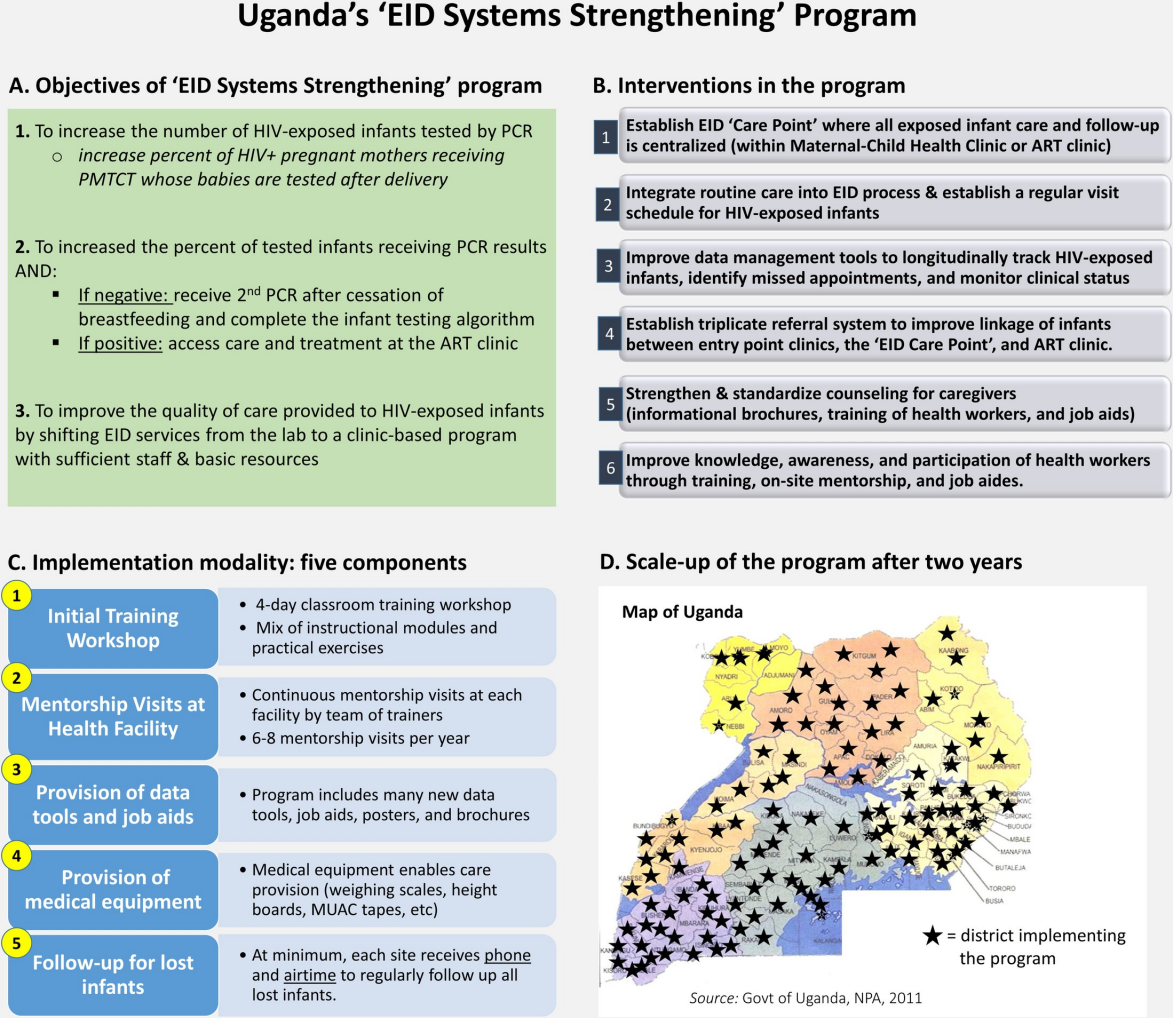
Uganda’s ‘EID Systems Strengthening’ program. A—Objectives of ‘EID Systems Strengthening’ program. B—Interventions in the program. C—Implementation modality: five components. D—Scale-up of the program after two years. Map source: Government of Uganda, National Planning Authority (NPA), 2011. Abbrev: EID = Early Infant Diagnosis, PMTCT = Prevention of Mother-to-Child Transmission, PCR = Polymerase Chain Reaction, MUAC = Mid-upper Arm Circumference.

In response to the identified system challenges at health facilities, the MOH, CHAI, and NGO stakeholders developed a set of integrated initiatives to improve testing, linkage, and retention of HEI and HIV+ infants, constituting the “EID Systems Strengthening” model (Fig 3 Panel). See S1 File for a comprehensive description of the program model and its component interventions. The model is comprised of 6 core initiatives: 1) establishment of an ‘EID Care Point’ at each facility where all care and follow-up of HEI is centralized, situated within either the ANC or ART clinic, 2) integration of regular clinical care for HEI along with a standardized monthly visit schedule, 3) new HEI data management and patient tracking tools to enable longitudinal follow-up of HEI, 4) a triplicate referral system to formally link infants between entry point clinics, the EID Care Point, and ART clinic, 5) informational brochures, job aids, and training to strengthen HWs’ counseling of HEI caregivers, and 6) training and mentorship of HWs to improve their knowledge and awareness on EID [10]. The program was implemented through an initial five-day classroom training of HWs followed by monthly mentorships at each health facility, coupled with medical equipment, data tools, job aids, brochures, and funds for phone calling and home visits to follow up lost HEI.

The MOH and CHAI piloted the ‘EID Systems Strengthening’ program at 21 facilities in 2010–11. After informal assessments at several MOH-CHAI pilot facilities showed gains in testing and retention of HEI, 8 NGOs supporting HIV service delivery (one per region) subsequently scaled up the program to all health facilities providing EID in Uganda through funding from the US President’s Emergency Plan for AIDS Relief (PEPFAR). The new model became the official national EID service delivery approach both for existing and newly trained EID sites, which has continued to the present time.

Barriers to testing and retention of HEI and HIV+ children in high burden settings have been extensively studied and well-documented through cohort studies from individual countries or hospitals [10–14], multi-country assessments [15, 16], and systematic reviews [17–21]. However, almost entirely missing from the literature are evaluations assessing the efficacy and feasibility of initiatives undertaken to tackle barriers to EID testing, linkage, and retention [22]. Such impact evaluations of targeted EID interventions can offer critical insights and evidence from ground-level implementation to inform development of optimal EID programmatic approaches and policies.

To assess the impact of Uganda’s ‘EID Systems Strengthening’ model, we conducted a mixed-methods evaluation study at 12 health facilities, consisting of a retrospective HEI cohort review and cross-sectional qualitative assessment of program implementation. In this paper, we present the study’s cohort findings, and compare them to the cohort outcomes reported in the 2010 ‘pre-intervention’ study. We then present the study’s qualitative findings on implementation fidelity, success factors, and implementation barriers. Finally, we discuss the model’s impact on testing and retention of HEI in Uganda, present and future implications, the model’s applicability to other countries, and key lessons learned.

## Materials and methods

### Study overview and health facility selection

The post-intervention evaluation study was conducted at 12 government health facilities in Uganda. The study consisted of 1) retrospective cohort review of HEI, aged 6 weeks to 18 months, who received a DBS test from June 2011 to May 2014 at the selected facilities, and 2) cross-sectional assessment of program implementation via semi-structured interviews of key informants (KIs) and structured clinic observation. Data collection was done in early 2016, allowing sufficient time after the cohort period (15 months) to follow all tested HEI through completion of the EID algorithm.

Health facilities were purposely selected to include a balance of facility levels and geographic regions, while taking into consideration previous HEI testing volumes and catchment population estimates. This evaluation included 1 ‘regional referral hospital’, 2 ‘general hospitals’ (district-level), 6 ‘health center IVs’, and 3 ‘health center IIIs’. See S2 File for a list of facilities and their regions. Most regions of the country were covered, and each facility was in a different district. For inclusion, selected facilities must have been providing PMTCT and EID services since 2007, and pediatric ART services since at least 2010.

### Retrospective cohort review: Data collection and analysis

In each health facility, data on HEIs were collected manually from many paper registers and data tools across several different clinics (Table 1), and inputted into a Microsoft Excel database. For the evaluation cohort, the minimum sample size of HEI required to detect statistical significance of changes from the baseline was 599 (for α = .05, power = 80%, and detectable proportion difference = .05, given baseline study cohort size of 1268). Based on the inclusion criteria, the HEI cohort’s sample size surpassed the minimum requirement.

**Table 1:** Outcome variables, health facility data points, and data sources for cohort study.

Outcome Variables	Health Facility Data Points	Primary Data Source(s)
⦁ Mean age at 1^st^ DBS test (all HEI and HIV+ only) ⦁ % tested at ≤ 2 months of age	⦁ Date of 1^st^ DBS test ⦁ Age at 1^st^ DBS test ⦁ 1^st^ PCR test Result	⦁ DBS Dispatch Book ⦁ HIV-Exposed Infant Register
⦁ % of tested HEI who received 1^st^ PCR results (all HEI and HIV+ only)	⦁ Did caregiver receive 1^st^ PCR results? ⦁ 1^st^ PCR test result	⦁ HEI Clinical Charts ⦁ PCR Result Forms ⦁ Appointment Books
⦁ % of HEI < 13 months and breastfeeding at 1^st^ DBS test [testing PCR-negative and receiving their results] who received confirmatory PCR test after cessation of breastfeeding	⦁ Age at 1^st^ DBS test ⦁ 1^st^ PCR test result ⦁ Feeding status at 1^st^ DBS test ⦁ If caregiver received 2^nd^ PCR result	⦁ HIV-Exposed Infant Register ⦁ HEI Clinical Charts ⦁ Appointment Book ⦁ PCR Result Forms
⦁ % of HIV+ pregnant women whose infants were brought for DBS testing after birth	⦁ HIV status of pregnant women in ANC ⦁ Gestational age / estimated delivery date ⦁ Mother of HEI ⦁ Date of DBS test	⦁ ANC Register ⦁ Exposed Infant Register ⦁ Referral Books
⦁ % HIV+ infants receiving results who were enrolled in care at an ART clinic ⦁ Age at enrollment at ART clinic	⦁ 1^st^ and 2^nd^ PCR test result ⦁ Whether caregiver received PCR result ⦁ Whether referred from EID Care Point ⦁ Whether enrolled at ART clinic and date ⦁ Age at 1^st^ ART clinic visit	⦁ Referral Forms ⦁ HIV-Exposed Infant Register ⦁ Pre-ART Register ⦁ ART Register
⦁ % HIV+ infants initiated on ART ⦁ % HIV+ infants active in care ⦁ Age at initiation on ART	⦁ Whether initiated on ART and date/age ⦁ ART Regimen, Clinical Stage, CD4 Results ⦁ Date of most recent visit	⦁ Pre-ART Register ⦁ ART Register ⦁ HIV/ART Card
⦁ Clinical care provision to HIV-exposed infants	⦁ Percent of all visits where HEI received growth monitoring, Cotrimoxazole, developmental milestone monitoring, clinical assessment	⦁ HEI Clinical Charts

HEI = HIV-Exposed Infants, DBS = Dried Blood Spot, PCR = Polymerase Chain Reaction, ANC = Antenatal Care Clinic, ART = Antiretroviral Therapy.

We used two measures to assess retention of tested HEI through the EID testing algorithm and care continuum: 1) whether 1^st^ PCR results were received by caregiver, and 2) whether breastfeeding HEI had a 2^nd^ confirmatory PCR after 12 months of age to diagnose postnatal HIV transmission. In measuring ART clinic retention among infants testing positive, the patient’s final retention status was defined as ‘active’ if s/he visited the clinic within the 3 months prior to November 2015. To assess HEI testing coverage, we collected data on linkage from PMTCT to EID services (distinct cohort of HIV+ pregnant women). The number of HIV+ pregnant mothers receiving PMTCT services represents the minimum number of HEI who would need a DBS test after birth. Therefore, the percent of HIV+ pregnant mothers in PMTCT who brought their infants after birth for DBS testing is a measure reflecting overall testing coverage of HEI. For clinical care, we assessed whether the standard national package of care was provided consistently to HEI as routine practice, from review of HEI charts corroborated with qualitative analysis of observation and KI interview data. We assessed counseling of caregivers qualitatively.

The cohort data were analyzed in Excel and SPSS. Hypothesis testing was done to assess statistical significance of changes in outcomes between the evaluation and baseline cohorts, with significance level α = .05. We conducted two-tailed z-tests for comparison of proportions and t-tests for comparison of means in two independent samples. See S3 File for data points from the cohort review.

### Comparison of outcomes between the pre- and post-intervention cohorts

We compared this evaluation study’s cohort outcome variables to the identical ones reported in the findings of the 2010 pre-intervention cohort study [10] to measure quantitative change (direction and magnitude). We didn’t conduct any secondary analysis of the underlying cohort data from the pre-intervention study; rather, we directly utilized the findings reported in that study’s paper as the set of baseline measures for comparison.

The outcome variables, underlying data points, and modality of facility-level data collection were nearly identical to those of the baseline study. The only exception was the indicator measuring confirmatory PCR testing of HEI who breastfed at initial DBS test: in the baseline cohort period, feeding practice of HEI at testing was rarely documented by HWs, which prevented a direct pre/post-intervention comparison. However, we calculated a proxy measure for this baseline indicator using the estimated 90% national prevalence of breastfeeding among HIV+ mothers during the pre-intervention period [23, 24]. The study could not quantitatively measure whether HEI presenting at a facility’s OPD or pediatric ward were being identified and linked to the EID Care Point due to inconsistent documentation in both cohort periods. We instead relied upon qualitative clinic observation data (same as baseline study’s method), while also assessing referral books’ utilization. We assessed whether the clinic care package was provided consistently to HEI, but couldn’t evaluate the quality of care within the study design.

The number and level of health facilities differed between the two studies. At the time of the baseline study cohort period (prior to 2010), EID was still relatively centralized. The highest level of HEI testing/diagnosis, care, counseling, data management, and tracking was provided at regional referral hospitals, whereas at lower-level facilities EID was generally limited to DBS sample collection and dispatch. Reflecting this context, the 2010 study’s cohort review was conducted at 3 regional referral hospitals. However, the ‘EID Systems Strengthening’ program interventions were designed to address HEI testing and retention gaps across all levels of Uganda’s health system. Further, in the two years between study cohorts, EID was rapidly scaled up to all facilities providing PMTCT in Uganda, with the ‘systems strengthening’ model replacing the previous lab-based program. Therefore, in order to conduct an impact evaluation of the ‘systems strengthening’ program reflective of its decentralized implementation, we sampled the HEI in our evaluation cohort from a cross-section of the 4 facility levels rather than from only the country’s highest-level hospitals, and included more facilities than in the baseline. Although differing from the baseline, this facility selection strengthened the generalizability (external validity) of the study findings for the evaluation cohort time period, thus providing a more current snapshot of Uganda’s overall HEI testing and retention situation, which in turn enabled our utilization of the findings to discuss ongoing challenges and future programmatic approaches in EID.

The difference in facility levels and quantity of facilities between the two cohorts may have adversely affected the validity of the data analysis specific to the pre/post-intervention outcome comparison. However, we believe the effect was minimal in this case. The study’s primary sampling unit was ‘HEI’ rather than ‘health facility’. Likewise, the ‘number of HEI’ was the primary sampling factor that affected validity of the pre/post comparison analysis (not ‘number of facilities).

Between the two cohort periods, there were no structural changes in how EID was implemented at the facilities beyond the ‘EID Systems Strengthening’ package of interventions. There were a few minor changes in national guidelines (e.g., longer breastfeeding period), but which didn’t have any direct bearing on study outcome variables. We cannot rule out possible effects from unknown confounding factors outside the scope of the ‘systems strengthening’ program (e.g. decrease in stigma over time). To mitigate this potential limitation, we focused our pre/post-intervention comparison narrowly on HEI testing and retention variables, which are mostly driven by facility-level ‘system’ factors rather than external factors. Importantly, our aim wasn’t to assess direct statistical causation, but to ascertain whether interventions likely contributed to any changes in outcomes.

### Qualitative assessment: Data collection, analysis, and triangulation

Semi-structured interviews with KIs (n = 51) and structured observations of EID implementation at health facilities aimed to provide an understanding of how the program was implemented at health facilities, which components were effective, implementation fidelity, and barriers and outstanding gaps. Topical guides were used for KI interviews and clinic observations.

KIs fell into 4 categories: health workers, national EID trainers, technical managers from implementing NGOs, and district health officials (Table 2). Each category of KI provided information from different perspectives. HW KIs provided experiences and insights as direct implementers in facilities, whereas the other 3 KI groups provided perspectives from a broader ‘big-picture’ lens. Interviews lasted 1 hour and were conducted in a private place.

**Table 2:** Key informant interviews: Category definitions, inclusion criteria, sample targets.

KI Category	Definition / Description	Minimum Criteria for Inclusion	Sample Target	Interview location
Health workers	Health workers involved in providing EID services at all reviewed facilities. Nurse, midwife, clinical officer, medical officer, lab technician, or related cadre.	Worked directly in EID service provision at a reviewed facility.	24 3 per hospital 2 per H/C IV 1 per H/C III	Health facilities
National EID trainers	A mix of personnel from government, NGOs, and private organizations. Trainers consist of nurses, doctors, counselors, or lab technicians.	Conducted mentorships at ≥ 3 reviewed facilities during review period. Possesses ≥ 1.5 years of experience as an EID trainer.	12 Selected from MoH database of master EID trainers	Institution they work in, or MoH offices
Implementing NGO technical managers	Technical managers from the NGO supporting and facilitating implementation of HIV services in each region. [Each region has 1 dedicated NGO for HIV implementation]	Senior technical manager from NGO supporting regional EID program implementation during review period (in a region with ≥ 1 reviewed facility)	5 1 per NGO/region: the senior-most technical manager	NGO’s regional office
District health staff	Staff within health division of local government, who are responsible for managing health services in the district. Includes DHO, Assistant DHOs, district program officers.	Involved in district-level management or administration of EID services during the review period (from districts containing ≥ 1 reviewed facility)	12 1–2 officials per district	District Health Office

EID = Early Infant Diagnosis, NGO = Non-Governmental Organization, DHO = District Health Officer.

‘Critical case’ purposive sampling was used. As each KI was someone who played an important role in the program, the interviews aimed to elicit rich and unique information based on the KI’s specific individual experiences, but under the assumption that those experiences were broadly similar to others of the same KI category. New KIs could be added until data saturation was achieved, as needed.

Study investigators conducted structured non-participant observation of EID implementation systems at the different units involved in EID within all 12 reviewed facilities (e.g., EID Care Point, ART clinic, referring clinics, lab). The observations captured how the ‘EID Systems Strengthening’ model was implemented at each facility, including whether the EID Care Point was located within the ANC or ART Clinic, where in the facility DBS samples were drawn (e.g., lab, EID care point, and/or entry point clinics), and the flow of patients, samples, test results and data within the health facility. Investigators assessed the extent to which the program interventions were being implemented.

The two qualitative methods were complementary. Whereas the observations provided direct examination of systems and practices, the interviews provided information and experiences from the lenses of key personnel involved in program implementation, mentorship, supervision, and/or management. Data from all interviews and observations were recorded in field note format and typed into Microsoft Word. The data were then imported into Atlas.ti, organized by topical area, and coded. Content analysis was conducted to bring out common themes in the frame of the research questions. The analyses from qualitative interview and observation data were then triangulated [25].

### Ethical approval

The study received ethical approval from the Mildmay Uganda Research and Ethics Committee. When inputting patient cohort data from health facility registers/charts into the study’s electronic database, investigators used unique identifiers in lieu of patient names to ensure confidentiality. Written informed consent was obtained from all KI interview participants, and consent for publication was obtained from HWs whose pictures are shown in S1–S3 Files.

## Results

### Retrospective cohort analysis

Our evaluation measured retention outcomes in a cohort of 707 HIV-exposed infants who received a DBS test at 12 health facilities in Uganda from June 2011 to May 2014. Positivity rate was 9.9% (67/675). PCR results weren’t found in clinic records for 32 tested HEI. We compared the evaluation cohort results to the pre-intervention baseline study, which tracked a cohort of 1,268 HEI receiving a DBS test at 3 regional referral hospitals from September 2007 –February 2009, of whom 244 tested HIV+ (19.2%) [10]. Table 3 reports the analysis of evaluation cohort data, and the comparison to baseline cohort outcomes.

**Table 3:** Cohort analysis and comparison to baseline study outcomes.

Indicators		Pre-Intervention	Post-Intervention	Comparing Baseline and Evaluation Outcomes		
		Baseline	Evaluation			
Retention and Survival of HIV+ Infants		Percent (x/N)	Percent (x/N)	Change (Magnitude)	95% CI	p value
Percent of HIV+ infants tested by PCR who are alive and active in care		**23.4%** (57/244)	**65.7%** (44/67)	**+ 42.3%**	0.32–0.53	p < .0001
	*Percent of HIV+ infants receiving PCR results*	**61.5%** (150/244)	**80.6%** (54/67)	**+ 19.1%**	0.08–0.30	p = .0018
	*Percent of HIV+ infants enrolled at ART Clinic (among those receiving results)*	**65.3%** (98/150)	**92.6%** (50/54)	**+ 27.3%**	0.16–0.39	p < .0001
	*Percent of HIV+ infants still alive and active in care (among those enrolled)* ^a^	**58.2%** (57/98)	**88.0%** (44/50)	**+ 29.8%**	0.16–0.43	p = .0001
**Retention of HEI through testing algorithm**		Percent (x/N)	Percent (x/N)	Change (Magnitude)	95% CI	p value
Percent of HEI who received 1^st^ PCR results ^b^		**56.6%** (718/1268)	**73.3%** (518/707)	**+ 16.6%**	0.13–0.20	p < .0001
Percent of breastfeeding HEI (receiving a negative 1^st^ PCR result) who had a 2^nd^ PCR		**20.4%** (48/235) ^c^	**54.5%** (192/352)	**+ 34.1%**	0.26–0.42	p < .0001
**Initiation of HIV+ Infants on ART (among those enrolled in care)**		Percent (x/N)	Percent (x/N)	Change (Magnitude)	95% CI	p value
Percent of eligible HIV+ infants initiated on ART after enrollment in chronic care ^d^		**36.0%** (27/75)	**92.0%** (46/50)	**+ 56.0%**	0.41–0.71	p < .0001
**Testing: PMTCT mothers whose infants are tested**		Percent (x/N)	Percent (x/N)	Change (Magnitude)	95% CI	p value
Percent of HIV+ pregnant women who brought their infants for testing after birth ^e^		**18.3%** (67/367)	**52.4%** (175/334)	**+ 34.1%**	0.27–0.41	p < .0001
**Age at Testing for HEI**		Percent (x/N)	Percent (x/N)	Change (Magnitude)	95% CI	p value
Percent of HEI tested by 2 months of age		**24.1%** (306/1268)	**52.6%** (368/700)	**+ 28.5%**	0.24–0.33	p < .0001
		Mean (SD, N)	Mean (SD, N)	Change (Magnitude)	95% CI	p value
Age (months) at first PCR among all HEI		**6.96** (5.9, 1268)	**4.21** (3.9, 700)	**- 2.75**	2.32–3.19	p < .0001
	Age (months) at first PCR for HIV+ only	**9.59** (7.3, 244)	**7.27** (5.2, 67)	**- 2.32**	0.77–3.88	p = .0018
	Age (months) at ART initiation (HIV+)	**16.07** (11.9, 27)	**9.82** (5.7, 42)	**- 6.25**	1.27–11.2	p = .0078
*COHORT DETAILS*:						
*Pre-Intervention HEI Cohort*: *3 Health Facilities*: *1268 HEI tested between September 2007 and Feb 2009*						
*Post-Intervention HEI Cohort*: *12 Health Facilities*: *707 HEI tested between June 2011 and May 2014*						

HEI = HIV-Exposed Infant; PCR = Polymerase Chain Reaction; CI = Confidence Interval, SD = Standard Deviation, P = Statistical Significance, N = Sample Size.

^a^ Defined as lost-to-follow-up if the HIV+ infant didn’t visit the ART clinic within the 3 months prior to Oct 31^st^ 2014.

^b^ Denominator includes missing PCR results (the 32 HEI without PCR results were defined as ‘not receiving results’).

^c^ This is a proxy measure estimate because HEI feeding status was documented inconsistently during the baseline period. The proxy measure was derived from baseline data on 2^nd^ PCR uptake (among HEI with negative 1^st^ PCR) and the national estimate of breastfeeding prevalence among HIV+ mothers during the baseline period.

^d^ Denominators were determined based on the national ART eligibility criteria in use during each review period.

^e^ In both baseline and evaluation periods, the denominator for the measure assessing testing coverage is a cohort of HIV+ pregnant women who received PMTCT at the health facility, distinct from the cohort of HIV-exposed infants receiving a DBS test during the review period.

#### Testing of HEI and linkage from entry points

We found a nearly three-fold increase in testing coverage of HEI: the percent of HIV+ pregnant mothers in PMTCT who brought their babies for DBS testing after birth increased from 18.3% (67/367) at baseline to 52.4% (175/334) (p < .0001). HEI were also tested at a much younger age (from mean age of 6.96 to 4.21 months, p < .0001). The percent receiving DBS test at ≤ 2 months increased from 24.1% (306/1268) at baseline to 52.6% (368/700) (p < .0001). In the evaluation cohort, the infection rate was much higher for HEI tested at > 2 months (15.9% positivity, 50/351) versus HEI tested at ≤ 2 months (4.8% positivity, 17/358) (p < .0001). In the post-intervention period, more entry point clinics (e.g., wards, OPD, immunization) were actively screening, identifying, and referring or escorting HEI to the facility’s EID service point as compared to baseline, from qualitative analysis. However, screening and linkage of HEI were still inconsistent at some facilities and some entry points.

#### Retention of HEI through the testing algorithm

HEI receiving 1^st^ PCR results—both positive and negative—increased from 56.6% (718/1268) to 73.3% (518/707) (p < .0001). Diagnosis of infection through breastfeeding improved. In the evaluation cohort, 93.7% (655/699) of HEI were breastfeeding at 1^st^ DBS test. Among HEI < 13 months, breastfeeding at 1^st^ DBS test, testing PCR-negative, and having received the negative result, 54.5% (192/352) received a 2^nd^ confirmatory PCR test after cessation of breastfeeding, with 12.0% positivity (23/192). In the baseline period, 18.4% (48/261) of HEI with a negative 1^st^ PCR and receiving their results had a confirmatory PCR test, but breastfeeding status of the HEI was rarely documented, preventing a direct pre/post comparison of 2^nd^ PCR uptake. However, a proxy measure for baseline HEI uptake of post-breastfeeding confirmatory PCR was estimated at 20.4% (48/235), calculated utilizing the estimated national breastfeeding prevalence among HIV+ mothers (90%). Using this pre-intervention baseline estimate, HEI uptake of confirmatory 2^nd^ PCR increased significantly from 20.4% to 54.5% (p < .0001).

#### Retention, linkage to care, and ART for HIV+ infants

Among infants testing HIV+ by PCR, retention in chronic care at an ART clinic increased from 23.4% (57/244) to 65.7% (44/67)—a nearly three-fold increase (p < .0001). Retention increased at each point in the EID cascade: HIV+ infants receiving their PCR results (61.5% to 80.6%, p = .0018), HIV+ infants enrolling in care at an ART clinic among those who received results (65.3% to 92.6%, p < .0001), and HIV+ infants active in care at the ART clinic (58.2% to 88.0%, p = .0001). Among HIV+ infants active in care, the percent successfully initiated on ART increased from 36% (27/75) to 92% (46/50) (p < .0001). Mean age at DBS test for infected infants decreased from baseline (from 9.59 to 7.27 months, p = .0018), corresponding to earlier mean age at ART initiation (from 16.07 to 9.82 months, p = .0078).

#### Clinical care of HEI

In contrast to the baseline period, clinical care was provided to HEI at each visit on a consistent basis at most reviewed facilities (Table 4). In the post-intervention period, more facilities carried out as routine practice growth monitoring, developmental assessment, immunization assessment and referral, and clinical assessment both for HIV indications and child well care.

**Table 4:** Comparison of clinical care provision between baseline and evaluation periods.

Care Indicators	# of facilities with clinical care consistently provided	
	Baseline Study Period (Pre-Intervention)	Evaluation Study Period (Post-Intervention)
**Cotrimoxazole provision to HEI**	3/7 facilities	12/12 facilities
**Growth monitoring** **(weight, height, mid-upper arm circum.)**	2/7 facilities	10/12 facilities
**Development assessment**	0/7 facilities	9/12 facilities
**Clinical assessment** **(HIV-specific and well care examination)**	5/7 facilities	12/12 facilities
**Immunization assessment and referral**	2/7 facilities	10/12 facilities
**DEFINITIONS:** *Care consistently provided*, *per each individual indicator* 1) Documented on HIV-exposed infant Clinical Charts at ≥ 80% of their visits, and 2) Care provided as routine practice at every clinic visit with rare exceptions (analysis of qualitative data) *Care inconsistently provided or not at all*, *per each individual indicator*: 1) Documented on HIV-exposed infant Clinical Charts at < 80% of their visits, and 2) Care provided occasionally or ad hoc, only when triggered for specific reason, or not provided at all (analysis of qualitative data)		

Results for care provision during the evaluation period were derived from analysis of qualitative data (direct observation of HIV-exposed infant clinic visits and interviews with key informants), with corroboration from documentation on clinical charts. During the baseline review period, exposed infants did not have clinical charts, and those findings were derived entirely from triangulation of qualitative data methods (health worker interviews and observation of clinic practices).

### Analysis of ‘EID Systems Strengthening’ program implementation

Triangulation of the qualitative data analyses from KI interviews and clinic observations yielded findings on implementation fidelity, relative effectiveness of different components of the ‘EID Systems Strengthening’ model, challenges, and outstanding gaps. The KI interviews included 24 HWs, 13 national EID trainers, 5 technical managers from regional NGOs, and 9 district health officials.

From the perspective of all 4 KI groups, the most impactful element of the ‘EID Systems Strengthening’ model was the establishment of an ‘EID Care Point’ at each health facility within its ANC or ART Clinic, coupled with a simplified, rational flow of caregivers, samples, results and data that reduced opportunities for patient loss. All reviewed facilities had successfully established an ‘EID Care Point’, staffed with an EID Focal Person and equipped with data tools, job aides, and medical equipment. KIs cited that the mere existence and functioning of the ‘EID Care Point’ was instrumental in shifting EID from a lab-focused program to a clinically focused chronic care service.

Facility-specific changes to clinic systems played an important role, with NGO technical staff and EID trainers providing numerous examples. For instance, several reviewed facilities operated their ART clinic only one day per week; most HIV+ infants receiving their PCR results were required to return on a separate day for clinic registration/enrollment, increasing the chance of patient loss. Through the ‘Systems Strengthening’ program, the facilities identified this as a problem, and adjusted their clinic system to ensure same-day enrollment at the ART clinic for all newly diagnosed patients.

Establishing a monthly visit schedule and integrating clinical care into EID visits facilitated regular interface between HEI caregiver and the health facility, which according to KIs, was a major reason why more HEI received the full package of services, including PCR results and confirmatory DBS tests. HW KIs noted that the new HEI Care Guidelines desk reference made it easier to quickly identify danger points for key indicators (e.g. low weight threshold) and take necessary actions (e.g. referral for therapeutic nutrition services). By second or third mentorship, EID trainer KIs noted that HWs, now equipped with a clinical chart for each HEI, had begun assessing for and acting upon longitudinal care trends that could indicate possible HIV infection (e.g. regression in infant’s developmental milestones).

We found that the new data management systems played an important role in evolving EID to a chronic care service. HWs reported that the longitudinal HEI Register enabled them to provide more comprehensive and holistic care because they could now readily view and monitor every HEI’s progression through the testing algorithm and care schedule. According to trainers and district health staff, simply possessing and inputting into a longitudinal register led HWs to conceive of EID as a chronic care program. However, appointment books were rarely used in most facilities, which trainers cited as a reason for defaulting HEI not being identified early or followed up.

The presence of triplicate referral books and mandate for HWs to use them contributed to HWs screening more proactively for HEI while completing the forms, according to NGO and district staff. HWs asserted that the HEI caregiver possessing the referral form helped remind them of their appointment at the destination clinic. The key element of the triplicate referral system was the ‘duplicate copy’ being sent directly from referring clinic to destination clinic (EID Care Point or ART Clinic), which would enable HWs to know if the referred infants didn’t materialize and then promptly follow up. However, we found this referral step wasn’t implemented across most sites—the duplicate copies weren’t filed in the designated binders at the destination clinics, nor acted upon to identify and recover referred infants.

A significant gap raised by HWs at every site was the lack of consistent funds to support phone calling or home visiting of defaulting infants. According to all KI groups, HWs weren’t motivated to utilize appointment books and triplicate referral forms to identify missing HEI largely because they lacked means to follow up.

The benefit of improving knowledge and awareness of HEI caregivers and HWs alike was heavily emphasized by KIs: HW and district officials reported that caregivers were better informed about the EID process and had greater awareness of the urgency of HIV diagnosis in infants. According to trainers and district officials, HWs in almost every unit in the facility were more knowledgeable about EID and more explicitly understood their roles in diagnosis and survival of HEI, leading to far more active participation, although some HWs and units were still lagging. KIs attributed improvements to standardized use of more persuasive and targeted messaging for HEI caregivers during counseling sessions, practical coaching of HWs on counseling techniques, and job aides for HWs to reference information. Engagement of all units of the health facility in mentorships and clearer definition of HW roles were also highlighted as important contributory factors. It was unclear if the informational brochures were beneficial, with HWs citing low literacy rates of caregivers even in local languages.

Without the monthly mentorship visits in the formative period after implementation, most program elements would not likely have taken hold or sustained, according to all KIs groups. Unlike the previous EID implementation model, health facilities received regular full-day mentorships after the initial classroom-style training. EID trainer KIs emphasized the vital role of the on-site mentorships in operationalizing the systems agreed upon at the initial workshop, and in ensuring that all departments were implementing in a coordinated way.

However, the frequency, intensity, and quality of mentorships decreased after the first year of implementation, contributing to an observed drop in quality of implementation over time. HWs and district officials asserted that lack of continuous mentorship over the long term made it difficult to sustain the system changes and initial program quality, particularly given frequent staff turnover and intra/inter-facility rotations.

Buy-in and direct involvement of the health facility’s administration emerged as the most critical determinant of whether and how well the new program was adopted early on. KIs noted that administration buy-in was especially critical since the program entailed changes to existing clinic systems (integrating new clinical service) and internal facility resource allocation (e.g. task-shifting). The program proved more durable at sites where facility in-charges took genuine ownership. According to trainers and NGO managers, despite involving and engaging facility administration in trainings and mentorships, inconsistent buy-in and lack of ownership were persistent challenges, hindering implementation quality and long-term sustainability.

## Discussion

Uganda’s ‘EID Systems Strengthening’ program revamped the EID service delivery model at health facilities. Our evaluation found that the model produced major gains in testing, retention, and linkages of HEI and HIV+ infants, compared to the pre-intervention baseline study results. Most critically, the percent of HIV+ infants tested by PCR who were initiated on ART and active in care increased from 23% to 66%, and for the first time, there was a dedicated place and mandate for comprehensive care of HEI. Intervention fidelity was strong for most program components.

Owing to the very large magnitude of positive change in almost all key outcome variables and high level of statistical confidence, we conclude that the ‘EID Systems Strengthening’ model had a sizeable effect on the study’s outcomes, driving the observed gains from the pre-intervention period in testing, linkage and retention for HEI and HIV+ infants. We do not infer direct causation because of the possible existence of unknown confounding factors independent of cohort characteristics and outside the program’s scope, as discussed earlier. However, even allowing for unknown external factors to have played a role, the sheer magnitude of positive change suggests a strong link between the interventions and observed outcomes. This latter point underpins our conclusion.

Even with the gains achieved through this set of system-focused interventions, the challenges of EID in Uganda remain daunting: retention in this study was still under 70% for HIV+ infants, and 48% of HEI born to HIV+ mothers in PMTCT were still not being tested. Our qualitative analysis revealed several gaps preventing the ‘EID Systems Strengthening’ program from achieving its full potential, which, if addressed, may increase its impact. Continuous mentorships at facilities after the first year of implementation are critical to consolidating and building upon the initial gains. The MOH should better monitor whether mentorships are being done, exerting pressure on donor agencies and NGO partners they support to consistently conduct mentorships over the long term. To enable follow-up of lost HEI, donors, NGOs and/or MOH need to provide consistent funding for phone calling and home visits. To address limited buy-in from health facility administration for system change, higher-level MOH engagement is needed. Perhaps performance in key EID metrics can be integrated into MOH evaluations of overall health facility performance.

A deeper challenge to the program’s long-term success is sustaining HW motivation and performance. Some program components weren’t consistently implemented, as HWs didn’t always see the tangible benefit of investing valuable time in new data management tools and clinic systems. However, if HWs regularly access data analysis from the MOH showing the retention outcomes of the HEI and HIV+ infants at their health facility, it would enable them to concretely connect their ‘input’ of work to their patients’ ‘outcomes’. For example, HWs seeing high attrition rates of their HEI might increase their motivation to regularly utilize appointment books and referral forms for follow-up of defaulters. Data-driven quality improvement (QI) is an approach that can be highly effective in improving HIV service delivery, in combination with other ‘systems engineering’ approaches [26]. QI approaches have been shown in several studies to improve different aspects of facility-level PMTCT implementation [27–30], and could be effective for EID in Uganda.

Building on the ‘Systems Strengthening’ approach, which continues to be Uganda’s current EID service delivery model, the country should prioritize deeper integration of services, aiming for a seamless continuum of coordinated HIV care for mother and baby. A promising step is an MOH pilot to expand the ‘EID Care Point’ into a ‘Mother-Baby Care Point’ at facilities, which enables provision of all HIV care for mother and baby at a single place with synchronized appointments, integrated mother/baby care, and streamlined follow-up [31]. In addition, loss of HIV+ pregnant women during the PMTCT process (antenatal, delivery, postnatal) has been a major driver of low HEI testing coverage in Uganda [32, 33]. To address this, Uganda’s MOH has integrated longitudinal tracking, appointment and referral systems, and higher quality of care standards into PMTCT implementation; an impact evaluation showed gains in antenatal retention, laboratory monitoring, facility deliveries, and postnatal care uptake [33]. Uganda could consider conducting PCR testing of HEI at birth (in addition to the PCR test at 6 weeks), which may contribute to earlier diagnosis and treatment for infected infants [34–36].

The success of the national EID program depends on timely sample-result transport and high-quality processing of samples at PCR testing labs, which have always been challenges in Uganda’s context. Uganda’s MOH has now shifted to a new hub-based sample transport system and consolidated the many regional testing labs into one centralized national lab. These initiatives have reduced sample-result turnaround time and program costs, while improving quality and efficiency of PCR testing [37, 38]. Uganda’s pilot of Short Message System (SMS) printers—which enables immediate transmission of PCR results to health facilities—produced further reductions in turnaround time at 10 facilities, suggesting potential for wider scale-up [39].

The global emergence of Point of Care (POC) EID technologies, which enables same-day diagnosis at health facilities, holds great promise for eliminating the loss point of HEI caregivers not receiving PCR results [40, 41]. POC EID tests have demonstrated comparable sensitivity to laboratory assays [34, 42, 43], and studies have demonstrated their effectiveness in accelerating diagnosis and treatment of HIV+ infants at country-level [44–46], and relative cost-effectiveness [47]. Uganda’s MOH has started piloting POC PCR testing for EID at selected health facilities. However, strong clinic systems will be vital to translate POC testing into improved HEI testing and retention outcomes.

### Applicability of the model to other countries and key takeaways

Despite significant progress in recent years, many countries continue to face challenges with loss of HEI and HIV+ infants throughout the EID cascade [48, 49]. Uganda’s approach may present a useful roadmap for countries considering different strategies to tackle persistent testing, linkage and retention challenges, which is now more critical than ever given the global policy shift to universal lifelong ART for HIV+ pregnant mothers.

**Fig 4:**
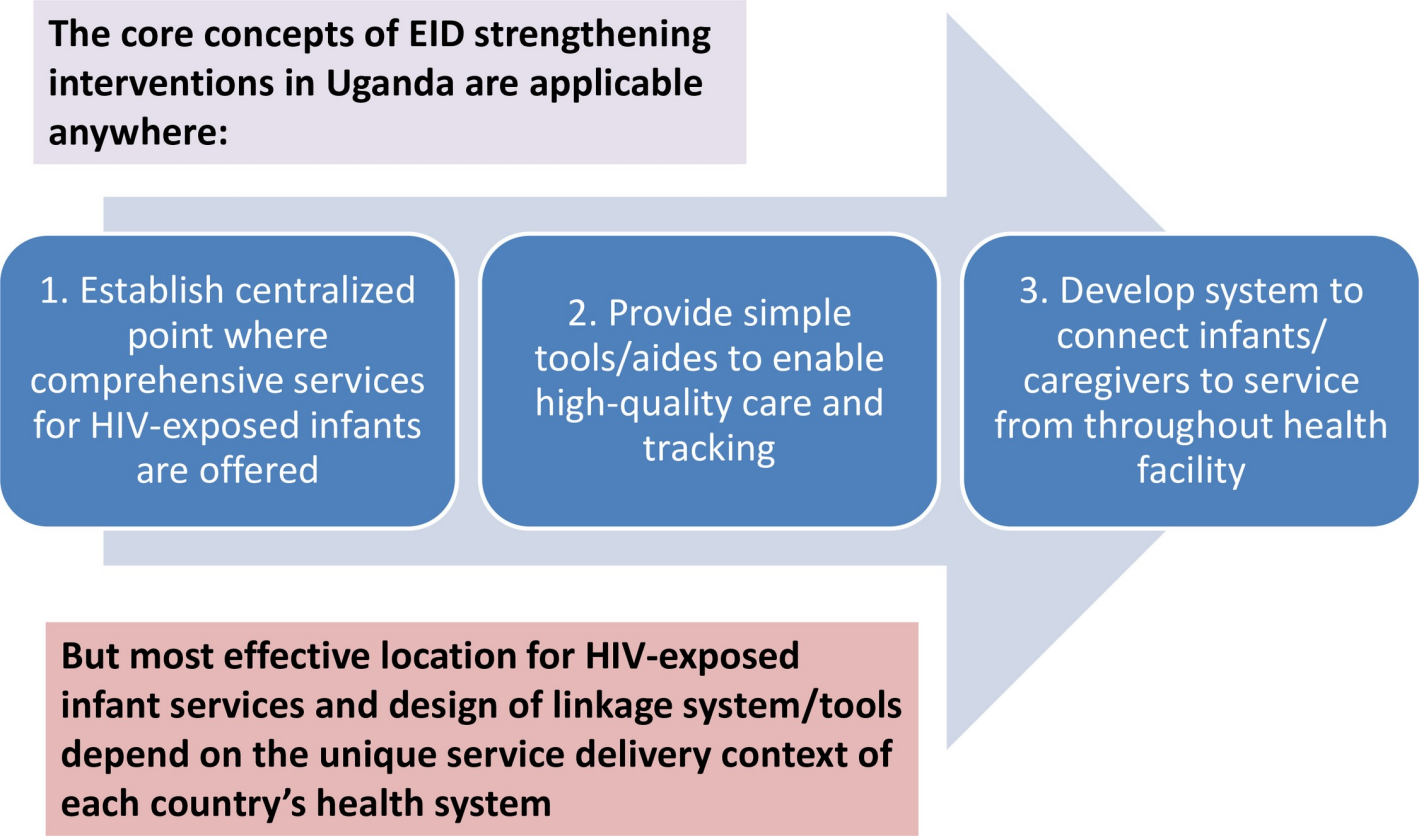
Applying Uganda’s “EID Systems Strengthening” model to other countries. Diagram showing how Uganda’s model can potentially be applied in countries facing similar challenges with retention of HIV-exposed infants.

The three core concepts of Uganda’s EID Systems Strengthening model are applicable to any country (Fig 4). First, establish a centralized point where comprehensive and high-quality services for HEI are offered. Second, provide the basic and essential tools to provide high-quality HEI care and longitudinal tracking at the service point. Third, develop a system to connect infants and caregivers to the service point from throughout the health facility. Each country then needs to determine the most effective location for delivery of HEI services within the health facility and the design of linkage/tracking systems; these choices would depend on the unique service delivery context of a country’s health system.

There are some useful takeaways from Uganda’s experience. In whatever EID implementation system a country develops, it is important to build in flexibility for each health facility to adapt a core model to fit its specific context—considering staffing, infrastructure, size, facility level, etc. In Uganda, this flexibility was inbuilt into the program’s roll out at each health facility. The staff designed their clinic flow, chose the location of their EID Care Point, decided where samples were collected and dispatched, and agreed on individual responsibilities. This enabled staff to “own” implementation of the program at their facility, which likely fostered the willingness among HWs to change ingrained systems and assume some additional workload, while increasing the project’s long-term sustainability.

Uganda’s experience underscores the benefits and importance of integration of HIV service provision at health facilities and synchronization of mother-baby care. To be successful, EID requires active participation by most, if not all, units of a health facility. The ‘EID Systems Strengthening’ approach proved effective because it established concrete mechanisms to facilitate coordination, collaboration and integration between units, which had previously been operating in silos.

The focus on improved care for HEI had a positive trickle-down effect on well-care for all children at Uganda’s health facilities, by exposing major gaps in quality and pushing higher standards of medical care for all pediatric patients (both HIV and non-HIV). Where possible, HIV-focused facility-level interventions in EID and other areas can be leveraged to strengthen overall quality of service delivery and health system capacity, which may help improve service integration [50, 51].

## Conclusion

Uganda’s approach has shown that targeting systemic facility-level bottlenecks to HEI retention, though potentially more time-intensive than the introduction of new rapidly-scalable technologies, is well worth the investment, as it can produce significant and self-sustaining program improvements, and at lower costs in the long-run.

## Supporting information

S1 File Description of Uganda’s ‘EID Systems Strengthening’ model. This reference document provides a more detailed description of the “EID Systems Strengthening” program model that was evaluated in this study.
(PDF)

S2 File List of health facilities and locations. (PDF)

S3 File Data points for cohort of HIV-exposed infants. (PDF)

## Abbreviations

HEI: HIV-Exposed Infant
HIV+: HIV-Positive
ART: Antiretroviral Therapy
EID: Early Infant Diagnosis (of HIV)
DBS: Dried Blood Spots
DNA: Deoxyribonucleic Acid
PCR: Polymerase Chain Reaction
ANC: Antenatal Clinic
PNC: Postnatal Clinic
OPD: Outpatient Department
MOH: Ministry of Health (of Uganda)
CHAI: Clinton Health Access Initiative
HW: Health Worker
MUAC: Mid-upper Arm Circumference
PMTCT: Prevention of Mother-to-Child Transmission (of HIV)
NGO: Non-governmental Organization
PEPFAR: (United States) President’s Emergency Plan for AIDS Relief
KI: Key Informant
DHO: District Health Officer
QI: Quality Improvement
SMS: Short Message System
POC: Point of Care
