# Supplementary material for: Uganda’s “EID Systems Strengthening” model produces significant gains in testing, linkage, and retention of HIV-exposed and infected infants: An impact evaluation
Source: PLoS One. 2021 Feb 4;16(2):e0246546. doi: 10.1371/journal.pone.0246546 (PMC7861549; doi:10.1371/journal.pone.0246546)
Supplement: S1 File — This reference document provides a more detailed description of the “EID Systems Strengthening” program model that was evaluated in this study. (PDF) [file pone.0246546.s001.pdf]

S1 File- Program Description
Uganda's "EID Systems Strengthening" Model

This reference document provides a detailed description of the "EID Systems Strengthening" program model that was evaluated in this study.

The document is meant to provide interested readers of this study with more detailed information about the program model, including each of the 6 interventions and how they targeted specific gaps in the previous EID service delivery model. This document may also be useful for program implementers and policy makers working to address testing and retention challenges in HIV Early Infant Diagnosis programs.

Table of Contents:

Program objectives and implementation modality…………………………………………………………. 2

Intervention 1: Establish an 'EID care point' in each facility where all testing, care, and follow-up of HIV-exposed infants is centralized………………………………………………………… 3

Intervention 2: Integrate clinical care into EID and establish a regular visit schedule………… 5

Intervention 3: Improve data management tools to longitudinally track HIV-exposed infants, identify missed appointments, and monitor clinical status…………………………………… 8

Intervention 4: Establish triplicate referral system to improve linkage of mothers & infants between entry point clinics, EID Care Point, and the ART Clinic……………………………… 9

Intervention 5: Strengthen & standardize counseling for caregivers of exposed infants…….. 10

Intervention 6: Improve knowledge, awareness and participation of health workers in EID and pediatric ART through training, on-site mentorship, and job aides…………………… 11

Program Objectives and Implementation Modality

Objectives of "EID Systems Strengthening" Program:

1) To increase the number of HIV-exposed infants tested by DNA PCR at health facilities

2) To increase the percent of tested infants receiving DNA PCR results AND:

- *If 1st PCR is negative*: receiving 2nd PCR after cessation of breastfeeding and complete the infant testing algorithm
- *If PCR is positive:* accessing care and treatment at the ART clinic

3) To improve the quality of care provided to HIV-exposed infants by shifting EID services from the lab to a clinic-based program with sufficient staff and basic resources

---

How the "EID Systems Strengthening" program is implemented:

1) Initial 4-day training workshop for health workers:
- Classroom setting— includes didactic sessions, practical exercises, and group work
- Multidisciplinary training team: medical officer, nurse, counselor, lab technician
- Each training workshop includes 3-5 health facilities
- Each health facility has 2-4 representatives from each clinic involved in EID activities

2) Mentorship visits to each health facility
- First mentorship visit is 2 weeks after workshop training to help set up new systems and kick-start implementation of all interventions
- Monthly mentorships
- Each mentorship is a full day and involves hands-on work with staff in all clinics

3) Provision of data tools, job aides, informational brochures
- Triplicate referral books, clinical charts, registers, DBS dispatch books, appointment books
- Clinical care guidelines (laminated desk reference)
- Posters/job aides for health workers: *EID testing algorithm, infant feeding guidelines, how to collect DBS samples, counseling messages, infant ART initiation guidelines*
- Brochures for caregivers of HIV-exposed infants (in English and local languages)
- Pre-organized binders for management of referral forms at the EID Care Point

4) Provision of medical equipment for care of HIV-exposed infants
- Weighing scales, height boards, MUAC tapes, head circumference tapes

5) Follow-up for infants who are lost-to-follow-up
- Mobile phone and airtime (phone credit)
- Funds for home visits to lost infants (transportation of health workers)

1. Establish an 'EID care point' in each facility where all testing, care, and follow-up of HIV-exposed infants is centralized. Streamline the flow of patients, DBS samples, test results, and data within the facility.

Previous systems and gaps: EID was a lab-based testing service rather than a chronic care service. EID was provided in an ad hoc and scattered way at health facilities. Clinical care, counseling, data management, follow-up for HIV-exposed infants were fragmented across different clinics within the health facility, with most poorly equipped to provide the minimum standard of services to HIV-exposed infants. Health facilities didn't have one, clear, well-equipped place where all HIV-exposed infants and their caregivers would go for registration and post-test counseling on the day of the DBS test, and then return monthly to receive test results, follow-up care, continuous counseling, and other services until completion of the EID process. Instead, infants often received care, counseling, tracking/follow-up at different places within the facility.

The "EID Care Point" model: Each health facility establishes an **"EID care point"** where all testing, care, counseling, data management, and follow up of HIV-exposed infants are centralized (**Figure S1**). The "EID Care Point" is integrated within either the Maternal Child Health or ART Clinic. It is equipped with sufficient space, medical equipment, prophylaxis, medications, data tools, job aides, and informational materials. It is staffed with at least 1 dedicated clinical staff.

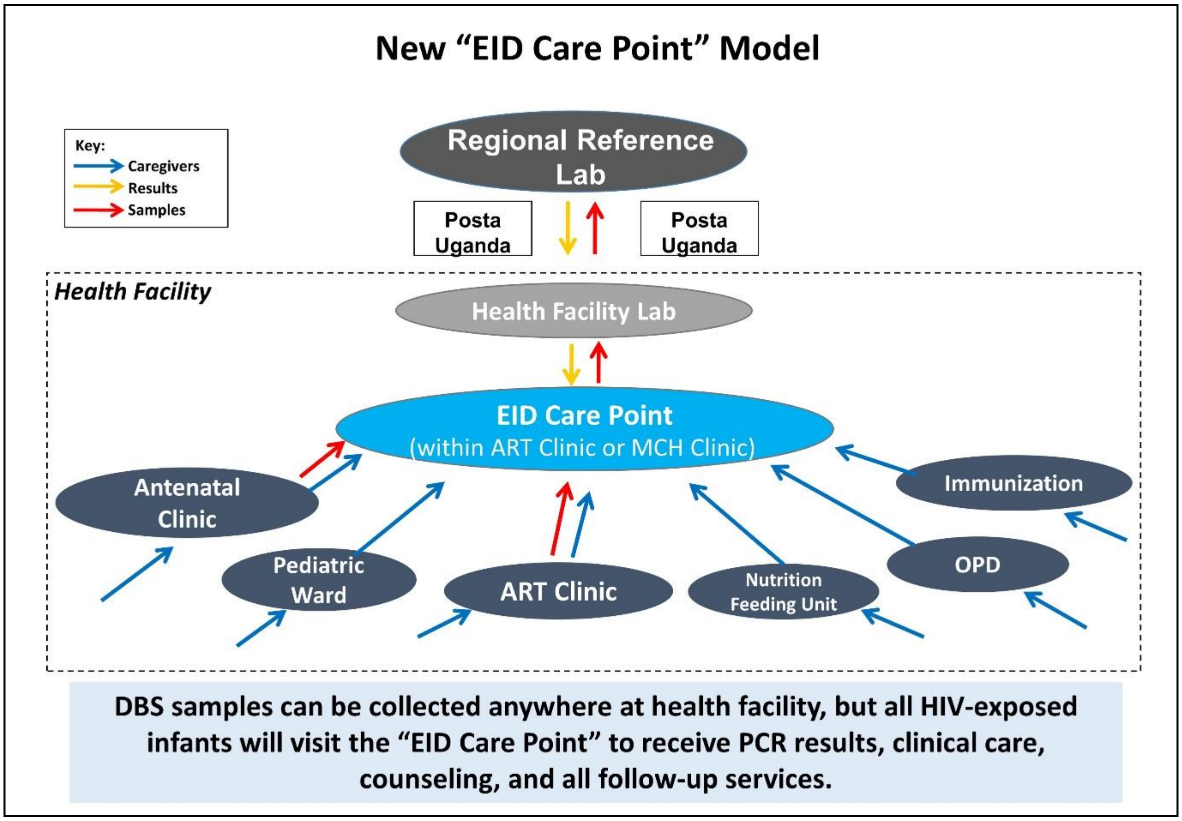

Figure S1: Flow chart showing the flow of patients, results, & samples in the "EID Care Point" model. HIV-exposed infants are identified at different points within a health facility, but all attend the EID care point for care, counseling, registration and follow-up starting at the first visit.

Clinic Flow: Each health facility adopts an EID clinic flow that works best for its context, but all HIV-exposed infants must receive the full complement of EID services at the "EID Care Point".

- When an HIV-exposed infant is identified at an entry point clinic, the caregiver is referred to the "EID care point" for registration, testing, care, and counseling. The caregiver and her infant then return to the "EID Care Point" for test results and all follow-up care visits.

- Facilities choose whether DBS samples will be collected at the entry point clinics, the "EID care point", and/or at the lab. The facility lab's primary role is to serve as the conduit for samples and test results between the "EID Care Point" and the external PCR testing laboratory.

Implementing the "EID Care Point" model: During the 'EID Systems Strengthening' workshop training, health workers are trained on the 'care point' model **(Figures S2 & S3)**. Staff and administration from each facility then decide on whether the EID care point will be located within the ART or MCH clinic, the clinic flow, which point(s) at the health facility will collect DBS samples, who will be the EID focal person(s), and the specific responsibilities of each staff member in implementation of EID services at the facility.

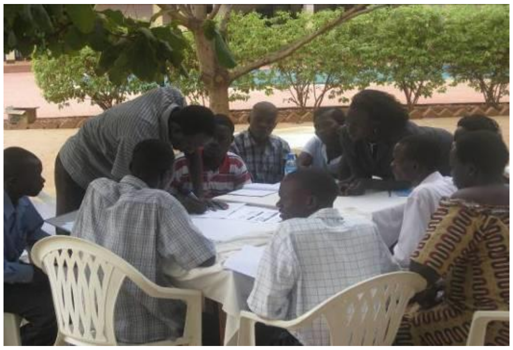

Figure S2: Staff from a hospital in northern Uganda discussing & re-designing their clinic system at the workshop training

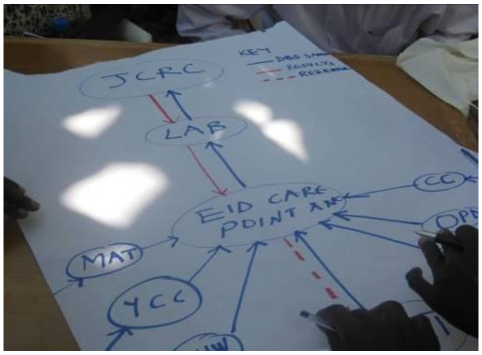

Figure S3: Staff at a hospital in east-central Uganda designing a flow chart depicting their new EID clinic system (during the training).

Figures S4 & S5 show a newly established EID Care Point, equipped with medical equipment for care provision, registers and clinical charts for patient tracking, referral books to link HIV+ infants to ART services, job aides, brochures, and a filing system to manage referral forms from entry point clinics.

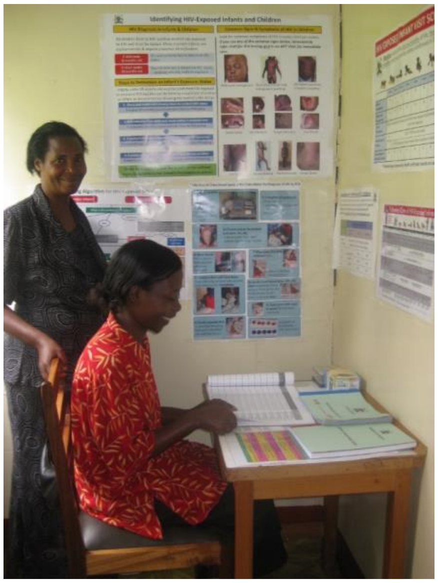

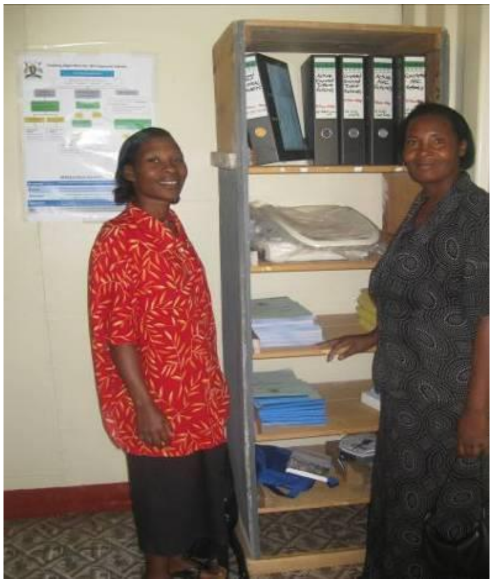

Figures S4 & S5: Newly established "EID Care Point" at Kitovu Hospital in western Uganda, with EID focal persons. It is integrated within the facility's MCH Clinic.

2. Integrate clinical care into the EID process and establish a regular visit schedule.

Previous systems: Clinical care was not provided to HIV-exposed infants as routine practice at most facilities. Counseling of caregivers was inconsistent and substandard. HIV-exposed infants did not attend the clinic on a regular basis: they only came for tests and retrieval of results. While PCR results were pending, infants who were HIV+ may have become symptomatic and required immediate ART. However, since the infants were not coming to the clinic regularly for care check-ups, clinical indicators of HIV infection may have been missed (e.g. weight loss, stunting). Opportunistic infections may have gone untreated until it was too late.

Integrating clinical care into EID:

The first step in establishing EID as a chronic care service is to ensure that HIV-exposed are attending and interfacing with the health facility on a regular basis, rather than only for DBS tests and PCR results.

The "EID system strengthening" model institutes a standardized visit schedule for HIV-exposed infants **(Figure S6)**. HIV-exposed infants attend the 'EID Care Point' monthly until 6 months of age, and every 3 months thereafter (more often if needed).

HIV-EXPOSED INFANT VISIT SCHEDULE

Monthly visits for the first six months of life, then every 3 months until final infection status confirmed at 18 months

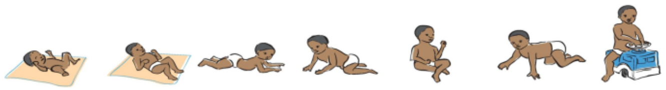

	6 wks	10 wks	14 wks	5 mo	6 mo	9 mo	12 mo	15 mo	18 mo
Immunization	x	x	x	-	x	x	x	-	x
Clinical Assessment	x	x	x	x	x	x	x	x	x
Growth and Development	x	x	x	x	x	x	x	x	x
Cotrimoxazole Prophylaxis	Start at 6 weeks and continue until infant has stopped breastfeeding and is determined to be HIV-negative								
Infant Diagnosis Testing	x PCR Test	x (if 1st PCR not yet done) 2nd PCR should be done 6 weeks after cessation of breastfeeding							x Antibody Test
Counseling and Feeding Guidance	x	x	x	x	x	x	x	x	x

Figure S6: Visit schedule for HIV-exposed infants, established as national policy

At each visit, exposed infants receive a minimum package of clinical care including:

- physical exam and clinical assessment
- growth monitoring
- developmental assessment
- immunization review and referral
- provision of prophylaxis
- linkage to other services as needed

Health workers receive medical equipment **(Fig S7)**: weighing scales, height boards, stadiometers, head circumference tapes, MUAC tapes, etc.

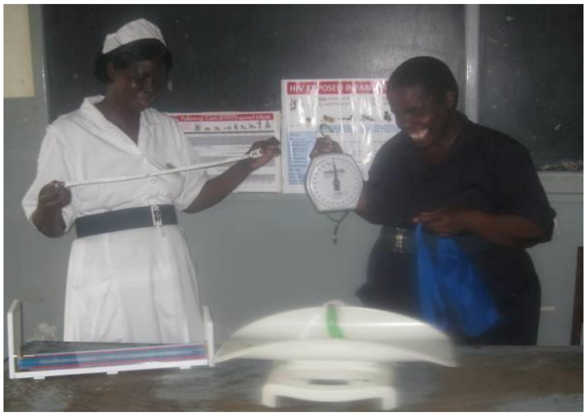

Figure S7: EID focal people at a hospital in eastern Uganda, having just received new medical equipment for clinical care of HIV-exposed infants

Health workers document care indicators on HIV-Exposed Infant Clinical Charts **(Figure S8).** The clinical charts enable health workers to holistically assess the health of the HIV-exposed infant at a given visit and act on any findings, but also to observe and monitor trends over time (such as progression of clinical symptoms, infections diagnosed at a previous visit not responsive to the treatment given, or slow growth rates).

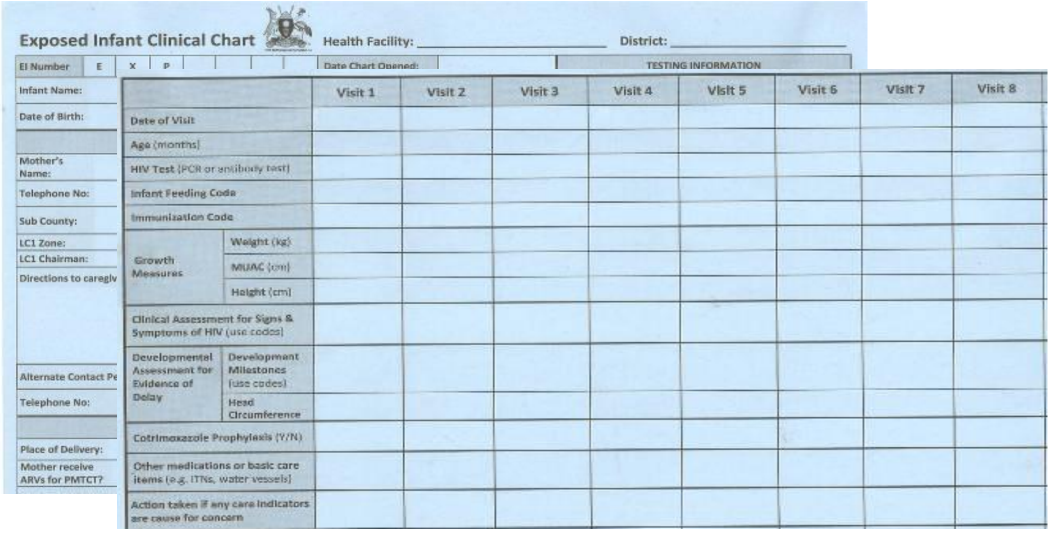

Exposed Infant Clinical Chart

Health Facility: ______________ District: ______________

EI Number | E | X | P | Date Chart Opened: | TESTING INFORMATION

Infant Name:
Date of Birth:
Mother's Name:
Telephone No:
Sub County:
LC1 Zone:
LC1 Chairman:
Directions to caregiv
Alternate Contact Pe
Telephone No:
Place of Delivery:
Mother receive ARVs for PMTCT?

		Visit 1	Visit 2	Visit 3	Visit 4	Visit 5	Visit 6	Visit 7	Visit 8
Date of Visit									
Age (months)									
HIV Test (PCR or antibody test)									
Infant Feeding Code									
Immunization Code									
Growth Measures	Weight (kg)								
	MUAC (cm)								
	Height (cm)								
Clinical Assessment for Signs & Symptoms of HIV (use codes)									
Developmental Assessment for Evidence of Delay	Development Milestones (use codes)								
	Head Circumference								
Cotrimoxazole Prophylaxis (Y/N)									
Other medications or basic care items (e.g. ITNs, water vessels)									
Action taken if any care indicators are cause for concern									

Figure S8: Clinical chart where care indicators are documented at each visit. This includes growth & development measures, feeding practice, immunizations, clinical assessment, immunizations, DBS tests, medications prescribed, and all actions taken at the visit based upon the clinical findings

---

Health workers are provided with a job aid containing guidelines for clinical care of HIV-exposed infants **(Figure S9).** The job aid enables health workers to identify danger signs and triggers action to address any problems. For example, after measuring weight and documenting it on the infant's clinical chart, the health worker checks the job aid to ensure that the infant's weight isn't below the danger point for his/her age.

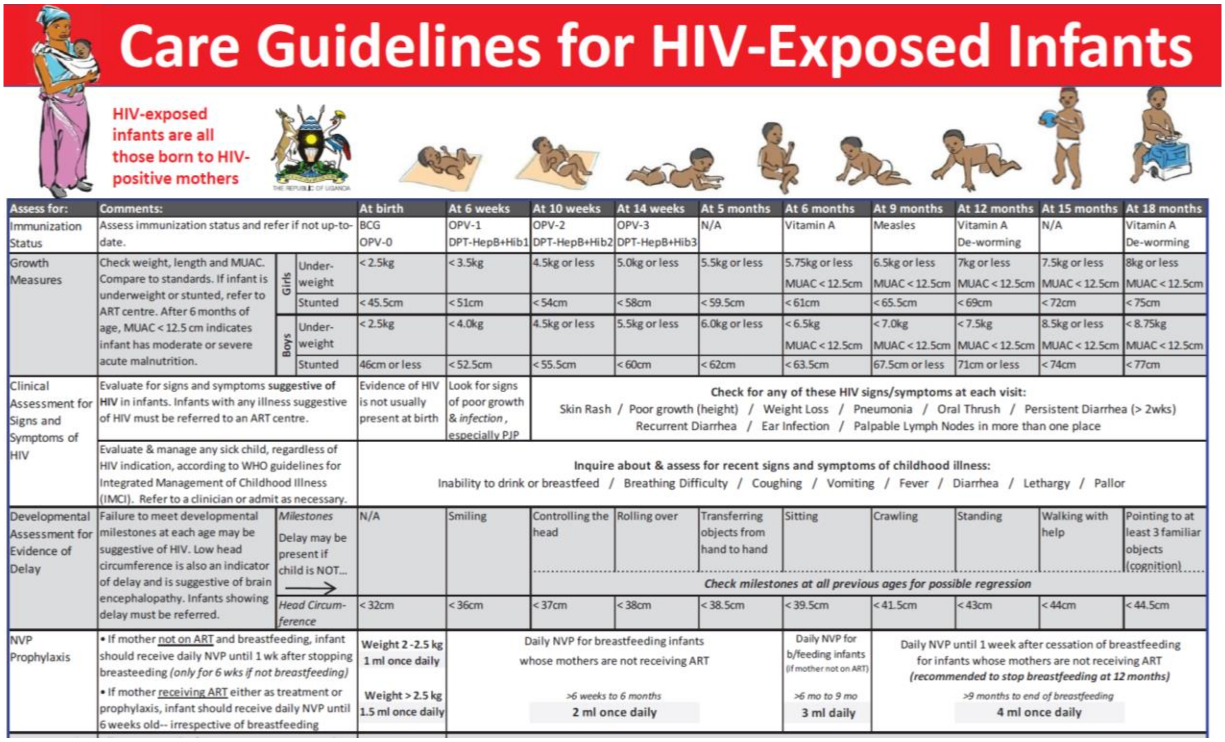

Care Guidelines for HIV-Exposed Infants

Assess for:	Comments:		At birth	At 6 weeks	At 10 weeks	At 14 weeks	At 5 months	At 6 months	At 9 months	At 12 months	At 15 months	At 18 months
Immunization Status	Assess immunization status and refer if not up-to-date.		BCG OPV-0	OPV-1 DPT-HepB+Hib1	OPV-2 DPT-HepB+Hib2	OPV-3 DPT-HepB+Hib3	N/A	Vitamin A	Measles	Vitamin A De-worming	N/A	Vitamin A De-worming
Growth Measures	Check weight, length and MUAC. Compare to standards. If infant is underweight or stunted, refer to ART centre. After 6 months of age, MUAC < 12.5 cm indicates infant has moderate or severe acute malnutrition.	Girls: Under-weight	< 2.5kg	< 3.5kg	4.5kg or less	5.0kg or less	5.5kg or less	5.75kg or less MUAC < 12.5cm	6.5kg or less MUAC < 12.5cm	7kg or less MUAC < 12.5cm	7.5kg or less MUAC < 12.5cm	8kg or less MUAC < 12.5cm
		Girls: Stunted	< 45.5cm	< 51cm	< 54cm	< 58cm	< 59.5cm	< 61cm	< 65.5cm	< 69cm	< 72cm	< 75cm
		Boys: Under-weight	< 2.5kg	< 4.0kg	4.5kg or less	5.5kg or less	6.0kg or less	< 6.5kg MUAC < 12.5cm	< 7.0kg MUAC < 12.5cm	< 7.5kg MUAC < 12.5cm	8.5kg or less MUAC < 12.5cm	< 8.75kg MUAC < 12.5cm
		Boys: Stunted	46cm or less	< 52.5cm	< 55.5cm	< 60cm	< 62cm	< 63.5cm	67.5cm or less	71cm or less	< 74cm	< 77cm
Clinical Assessment for Signs and Symptoms of HIV	Evaluate for signs and symptoms **suggestive of HIV** in infants. Infants with any illness suggestive of HIV must be referred to an ART centre.		Evidence of HIV is not usually present at birth	Look for signs of poor growth & *infection*, especially PJP	**Check for any of these HIV signs/symptoms at each visit:** Skin Rash / Poor growth (height) / Weight Loss / Pneumonia / Oral Thrush / Persistent Diarrhea (> 2wks) Recurrent Diarrhea / Ear Infection / Palpable Lymph Nodes in more than one place							
	Evaluate & manage any sick child, regardless of HIV indication, according to WHO guidelines for Integrated Management of Childhood Illness (IMCI). Refer to a clinician or admit as necessary.		**Inquire about & assess for recent signs and symptoms of childhood illness:** Inability to drink or breastfeed / Breathing Difficulty / Coughing / Vomiting / Fever / Diarrhea / Lethargy / Pallor									
Developmental Assessment for Evidence of Delay	Failure to meet developmental milestones at each age may be suggestive of HIV. Low head circumference is also an indicator of delay and is suggestive of brain encephalopathy. Infants showing delay must be referred.	*Milestones* Delay may be present if child is NOT... →	N/A	Smiling	Controlling the head	Rolling over	Transferring objects from hand to hand	Sitting	Crawling	Standing	Walking with help	Pointing to at least 3 familiar objects (cognition)
					Check milestones at all previous ages for possible regression							
		Head Circum-ference	< 32cm	< 36cm	< 37cm	< 38cm	< 38.5cm	< 39.5cm	< 41.5cm	< 43cm	< 44cm	< 44.5cm
NVP Prophylaxis	• If mother not on ART and breastfeeding, infant should receive daily NVP until 1 wk after stopping breasteeding *(only for 6 wks if not breastfeeding)*<br>• If mother receiving ART either as treatment or prophylaxis, infant should receive daily NVP until 6 weeks old-- irrespective of breastfeeding		**Weight 2 -2.5 kg 1 ml once daily**<br>**Weight > 2.5 kg 1.5 ml once daily**	Daily NVP for breastfeeding infants whose mothers are not receiving ART<br>*>6 weeks to 6 months*<br>**2 ml once daily**				Daily NVP for b/feeding infants (if mother not on ART)<br>*>6 mo to 9 mo*<br>**3 ml daily**	Daily NVP until 1 week after cessation of breastfeeding for infants whose mothers are not receiving ART ***(recommended to stop breastfeeding at 12 months)***<br>*>9 months to end of breastfeeding*<br>**4 ml once daily**			

Figure S9: Job aid enabling health workers to interpret the clinical measures recorded on the infant's clinical chart, identify any abnormalities in growth and development, and take prompt action

How the clinical care interventions work together: **Figure S10** shows how the visit schedule, clinical charts, and clinical care guidelines work together to facilitate chronic care provision for HIV-exposed infants.

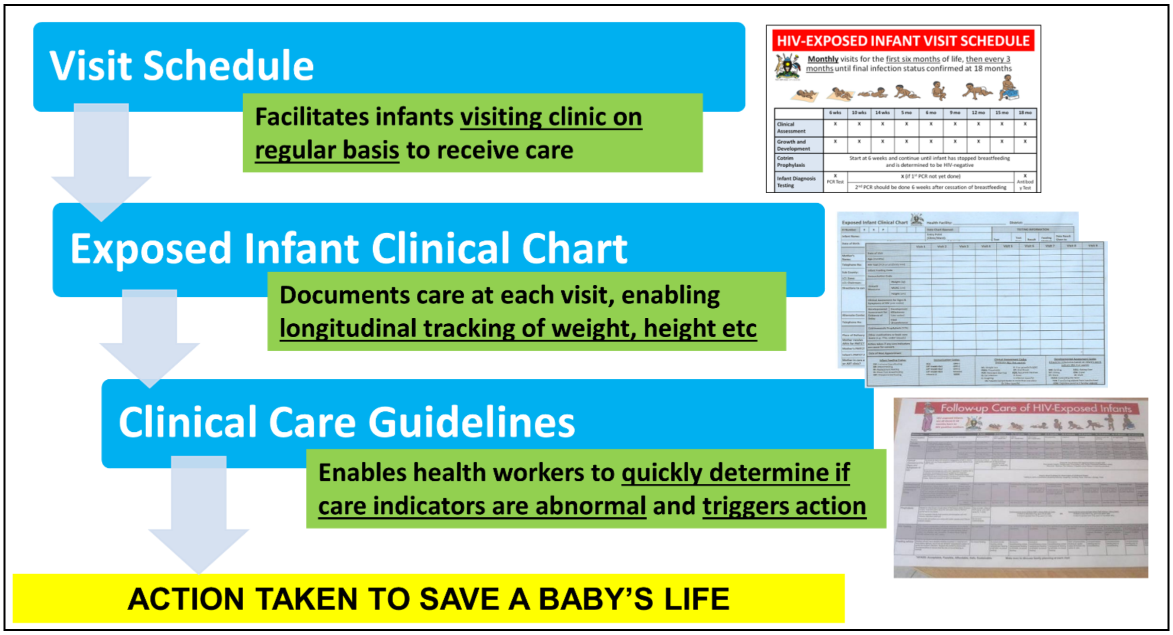

Figure S10: Interventions to integrate clinical care into the EID program at each health facility:
1) creation of a standardized visit schedule,
2) provision & utilization of clinical charts, and
3) establishment of clinical care guidelines for HIV-exposed infants

---

Training on clinical care: During the workshop training and subsequent on-site mentorship visits, health workers are trained on provision of clinical care to HIV-exposed infants (both theory and practical).

Health workers are also trained on how to use the clinical charts and care guidelines job aid in combination to identify warning signs, integrate findings from different assessments to make differential diagnoses (e.g. growth measures, developmental assessment, feeding practices), and trigger actions that may save an HIV-exposed infant's life.

3. Improve data management tools to longitudinally track HIV-exposed infants, identify missed appointments, and monitor clinical status.

Gaps in previous systems:

- The previous EID data register was a "one-visit" patient management tool— it did not capture any follow-up visits after the infant's DBS test. This limited the ability of health workers to track an exposed infant's progress through the testing algorithm. Health workers could not tell from the register whether the infant had received 1st PCR results, whether still breastfeeding, when due for 2nd PCR, etc.
- There was no system to document and manage follow-up appointments for HIV-exposed infants, preventing quick identification and follow-up of infants who did not attend the clinic.
- HIV-exposed infants lacked individual patient files, so there was no place to document the caregiver's phone contact and directions to their home. There was no place to document clinical care indicators.
- Health workers often resorted to making homemade registers to capture info, increasing their workload.

New tracking tools: Health workers use 3 new data tools that follow HIV-exposed infants longitudinally through the testing algorithm, enable identification of missed appts, and tracks clinical care indicators at each visit.

1. **HIV-Exposed Infant Register** (Figure S11): A longitudinal register that captures critical info about each follow-up visit, and tracks exposed infants through completion of the testing algorithm and care schedule. It contains sections for 1st PCR, 2nd PCR, confirmatory tests, and follow-up visits.
2. **Patient Appointment Book** (Figure S12): A tool that enables immediate recognition of missed appointments and triggers follow-up. It enables better management of daily patient volumes.
3. **Exposed Infant Clinical Chart** (Fig S8): Comprehensive patient record for each exposed infant. It captures care indicators at each visit and has contact information to enable follow-up of defaulters.

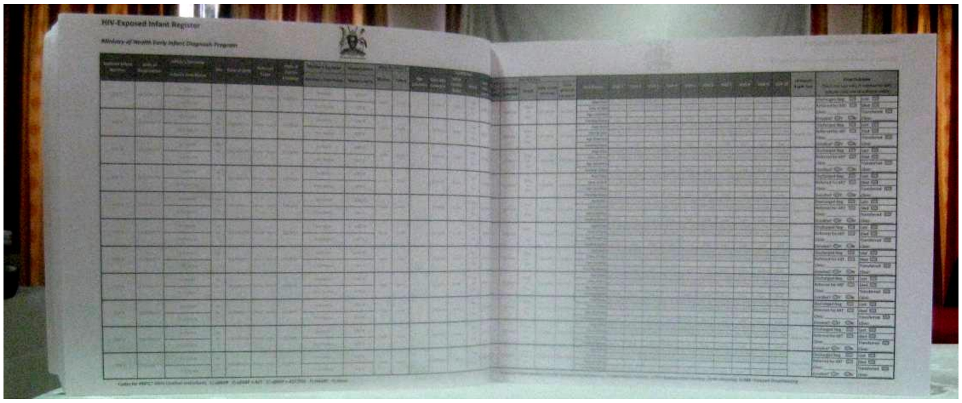

Figure S11: HIV-Exposed Infant Register

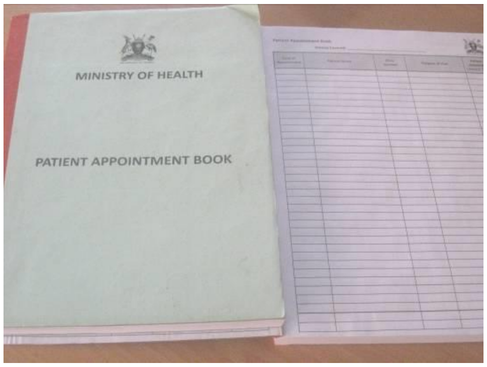

Figure S12: Appointment Book for HIV-Exposed Infants

4. Establish a referral system to improve linkage of mothers & infants between entry points clinics, EID Care Point, and ART Clinic.

<u>*Gaps in previous systems:*</u> Many HIV-exposed infants identified at the health facility were not reaching the EID service point and getting tested. Health workers at entry point clinics (e.g. wards, outpatient department) would verbally refer HIV-exposed infants to the EID testing point. However, the EID testing point didn't know there was an exposed infant who had been referred, and wasn't able to follow-up if the infant did not attend. Similarly, HIV+ infants were verbally referred to the ART clinic after receiving their PCR results, but the ART clinic did not know to expect an HIV+ infant and could not follow up.

<u>*Triplicate Referral System:*</u> **Figure S13** shows how the triplicate referral system is used to link HIV-exposed infants and HIV+ individuals from referral clinic to destination clinic within a health facility.

- Health workers complete the 'referral form' at the point of referral:
  - first copy is provided to the caregiver
  - second copy is brought directly by the health worker to the destination clinic
  - third copy remains at the referring clinic.
- If the referred infant does not attend the destination clinic, health workers follow up by phone or home visit (contact info is recorded on the referral form).
- The referral system also enables referral between different health facilities (e.g. transfers).

The triplicate referral forms are primarily used to:

a. refer HIV+ pregnant mothers (receiving antenatal care) to bring their infants to the 'EID Care Point' for testing and care *6 weeks after delivery*

b. refer HIV-exposed infants from entry point clinics to the 'EID Care Point' for testing & care

c. refer HIV+ infants from the 'EID Care Point' to the ART clinic for care and treatment

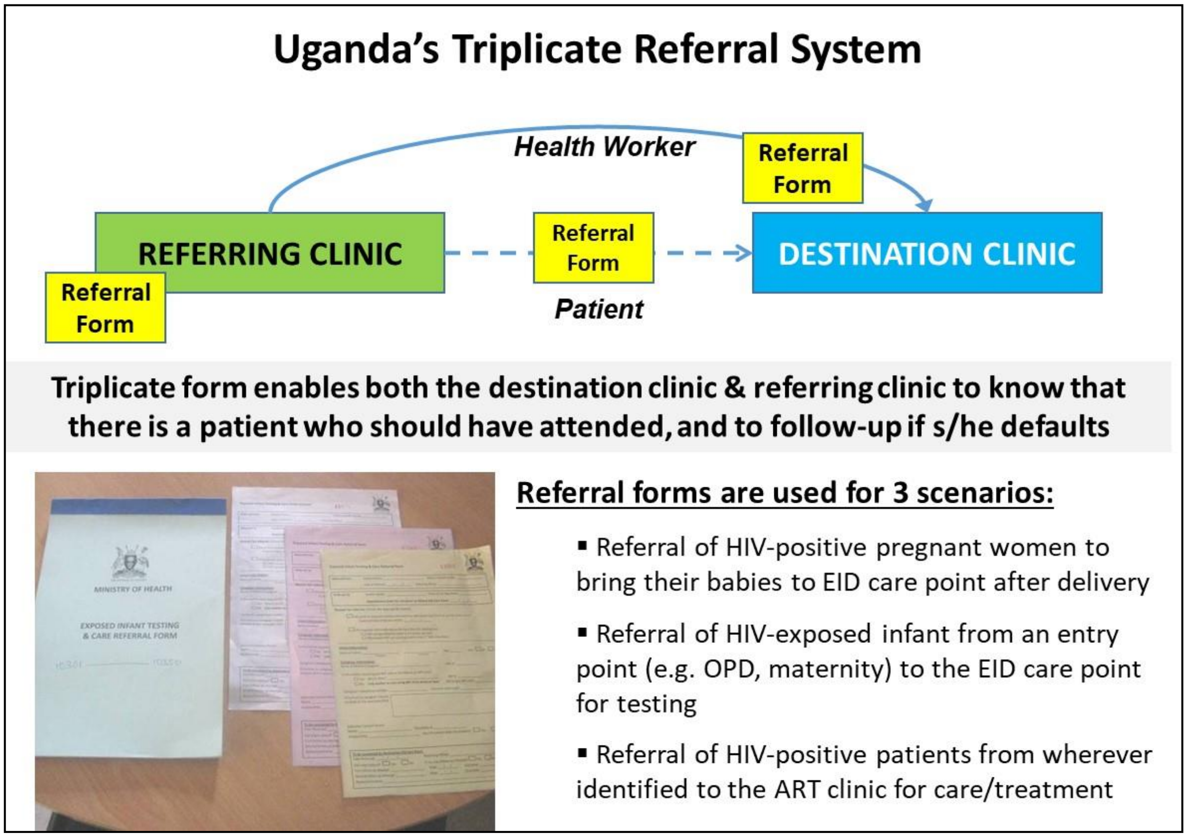

Figure S13: Triplicate referral system links HIV-exposed & infected infants to EID & ART services

5. Strengthen and standardize counseling for caregivers of HIV-exposed infants.

Poor counseling contributes to the loss of HIV-exposed infants at every stage in the EID process. This program includes 3 components to improve knowledge and awareness of caregivers of HIV-exposed infants.

- **Take-home brochures:** **(Figure S14)** Pamphlets given to caregivers of exposed infant provide key informational and sensitization messages, and reinforce counseling provided during the clinic visits.
- **Training & mentorship of health workers** **(Figure S15):** During the initial workshop training, health workers learn effective counseling techniques, strengthen counseling skills through practical exercises, and master the key messages for caregivers. This is reinforced during monthly mentorships.
- **Job Aid/Poster:** A job aid will be posted at every clinic in the health facility to guide health workers on what counseling messages to communicate to caregivers of exposed infants.

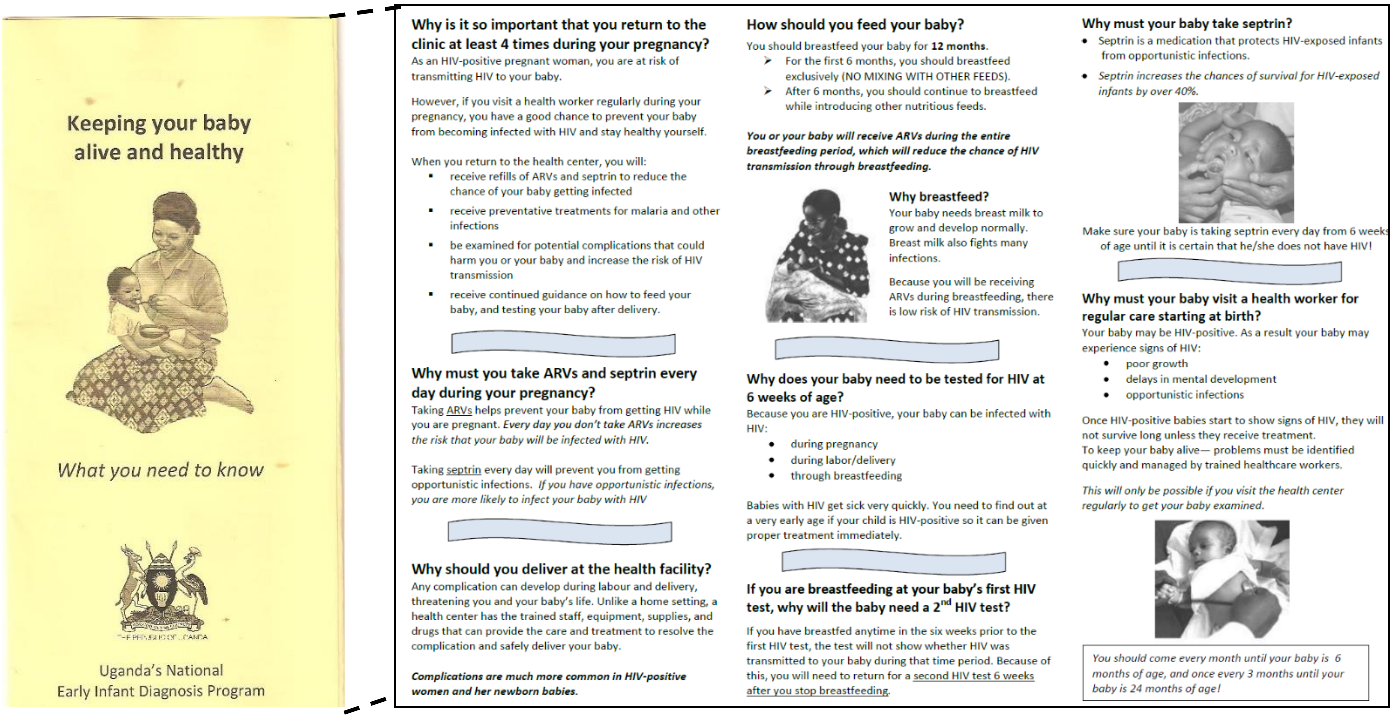
Keeping your baby alive and healthy

What you need to know

Uganda's National Early Infant Diagnosis Program

Why is it so important that you return to the clinic at least 4 times during your pregnancy?
As an HIV-positive pregnant woman, you are at risk of transmitting HIV to your baby.

However, if you visit a health worker regularly during your pregnancy, you have a good chance to prevent your baby from becoming infected with HIV and stay healthy yourself.

When you return to the health center, you will:
- receive refills of ARVs and septrin to reduce the chance of your baby getting infected
- receive preventative treatments for malaria and other infections
- be examined for potential complications that could harm you or your baby and increase the risk of HIV transmission
- receive continued guidance on how to feed your baby, and testing your baby after delivery.

Why must you take ARVs and septrin every day during your pregnancy?
Taking ARVs helps prevent your baby from getting HIV while you are pregnant. *Every day you don't take ARVs increases the risk that your baby will be infected with HIV.*

Taking septrin every day will prevent you from getting opportunistic infections. *If you have opportunistic infections, you are more likely to infect your baby with HIV*

Why should you deliver at the health facility?
Any complication can develop during labour and delivery, threatening you and your baby's life. Unlike a home setting, a health center has the trained staff, equipment, supplies, and drugs that can provide the care and treatment to resolve the complication and safely deliver your baby.

Complications are much more common in HIV-positive women and her newborn babies.

How should you feed your baby?
You should breastfeed your baby for **12 months**.
- For the first 6 months, you should breastfeed exclusively (NO MIXING WITH OTHER FEEDS).
- After 6 months, you should continue to breastfeed while introducing other nutritious feeds.

You or your baby will receive ARVs during the entire breastfeeding period, which will reduce the chance of HIV transmission through breastfeeding.

Why breastfeed?
Your baby needs breast milk to grow and develop normally. Breast milk also fights many infections.

Because you will be receiving ARVs during breastfeeding, there is low risk of HIV transmission.

Why does your baby need to be tested for HIV at 6 weeks of age?
Because you are HIV-positive, your baby can be infected with HIV:
- during pregnancy
- during labor/delivery
- through breastfeeding

Babies with HIV get sick very quickly. You need to find out at a very early age if your child is HIV-positive so it can be given proper treatment immediately.

If you are breastfeeding at your baby's first HIV test, why will the baby need a 2nd HIV test?
If you have breastfed anytime in the six weeks prior to the first HIV test, the test will not show whether HIV was transmitted to your baby during that time period. Because of this, you will need to return for a second HIV test 6 weeks after you stop breastfeeding.

Why must your baby take septrin?
- Septrin is a medication that protects HIV-exposed infants from opportunistic infections.
- *Septrin increases the chances of survival for HIV-exposed infants by over 40%.*

Make sure your baby is taking septrin every day from 6 weeks of age until it is certain that he/she does not have HIV!

Why must your baby visit a health worker for regular care starting at birth?
Your baby may be HIV-positive. As a result your baby may experience signs of HIV:
- poor growth
- delays in mental development
- opportunistic infections

Once HIV-positive babies start to show signs of HIV, they will not survive long unless they receive treatment.
To keep your baby alive— problems must be identified quickly and managed by trained healthcare workers.

This will only be possible if you visit the health center regularly to get your baby examined.

You should come every month until your baby is 6 months of age, and once every 3 months until your baby is 24 months of age!

Figure S14: Informational brochures for caregivers of HIV-exposed infants— in both English and local languages

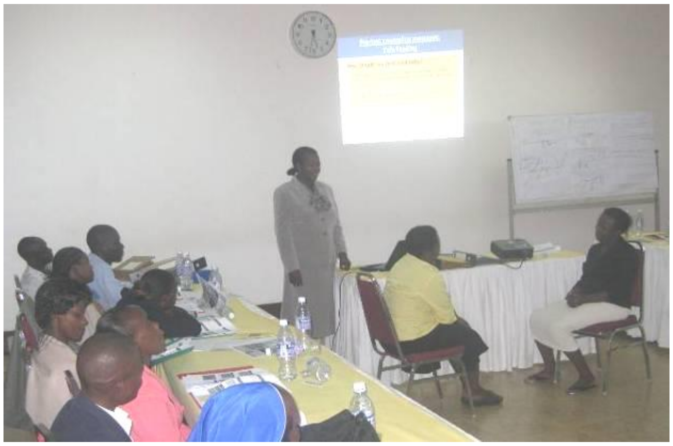

Figure S15: Health workers role-playing counseling scenarios at the training

6. Improve knowledge, awareness and participation of health workers in EID and pediatric ART through training, on-site mentorship, and job aids.

Previous gaps: Many health workers lacked adequate knowledge of EID and pediatric HIV, including how to screen for HIV exposure, collection of DBS samples, clinical care standards, counseling messaging, testing and feeding algorithms, and signs/symptoms of pediatric HIV infection. In addition, many health workers were not actively participating in EID program implementation- especially in screening, identification and referral of HIV-exposed infants encountering the health facility at entry point clinics.

Interventions to improve health worker knowledge and awareness about EID:

1. Initial classroom 'workshop' training
2. Monthly mentorship visits to each health facility
3. Job aides and posters for health workers **(Figure S16)**

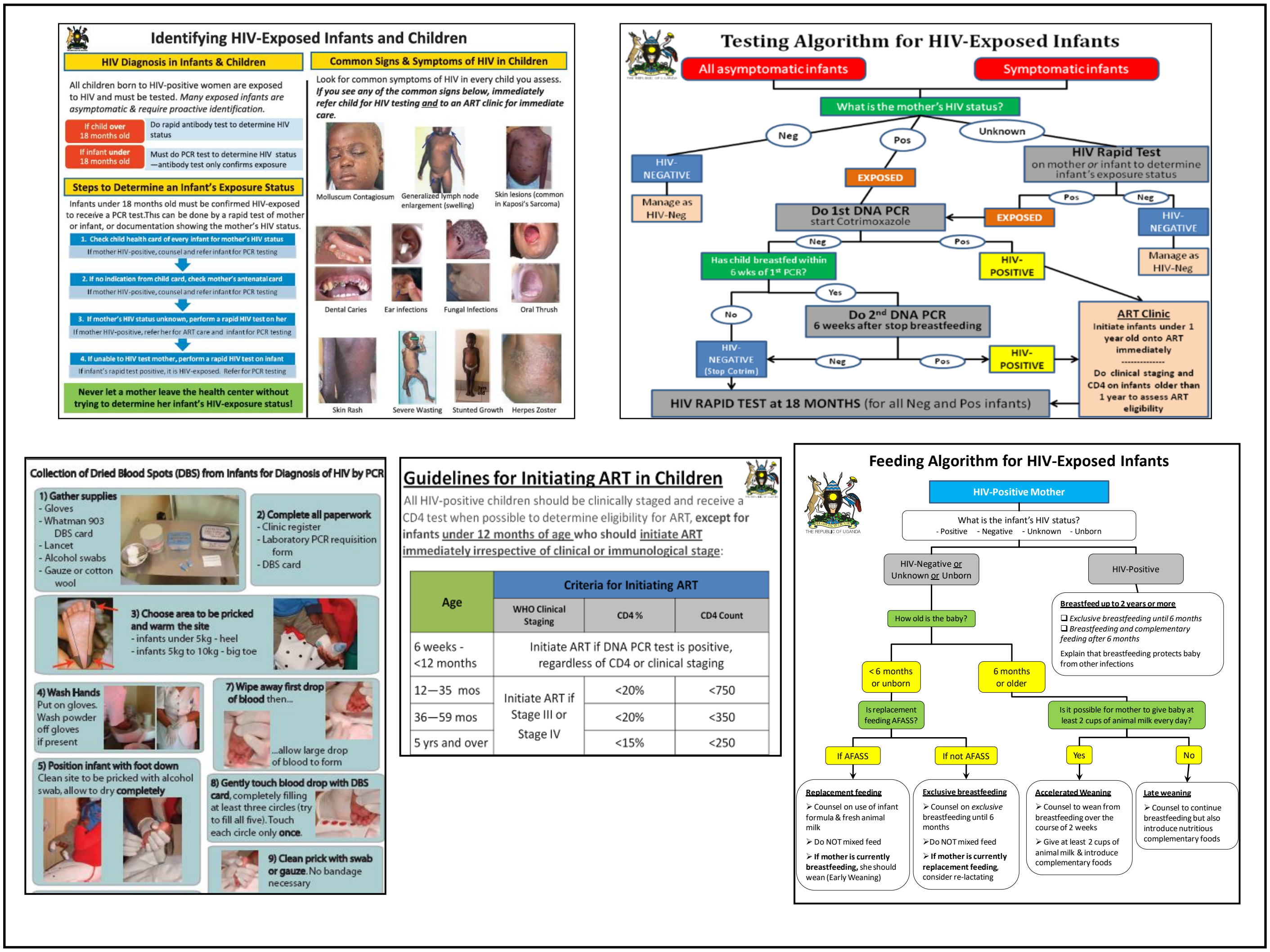

Figure S16: Posters & job aides posted at all clinics involved in EID implementation at the health facility
