# Supplementary material for: Uganda’s “EID Systems Strengthening” model produces significant gains in testing, linkage, and retention of HIV-exposed and infected infants: An impact evaluation
Source: PLoS One. 2021 Feb 4;16(2):e0246546. doi: 10.1371/journal.pone.0246546 (PMC7861549; doi:10.1371/journal.pone.0246546)
Supplement: S2 File — (PDF) [file pone.0246546.s002.pdf]

S2 File: List of Health Facilities and Locations

Sites of EID Evaluation Study				
**#**	**Health Facility Name**	**Level**	**Region**	**District**
1	Anaka Hospital	General Hospital	North	Nwoya
2	Bobi Health Center III	Health Centre III	North	Gulu
3	Ayipe Health Center III	Health Centre III	West Nile (Northwest)	Koboko
4	Bondo Health Center III	Health Centre III	West Nile (Northwest)	Arua
5	Nebbi Hospital	General Hospital	West Nile (Northwest)	Nebbi
6	Midigo Health Center IV	Health Centre IV	West Nile (Northwest)	Yumbe
7	Karenga Health Center IV	Health Centre IV	Northeast	Kaabong
8	Namukora Health Center IV	Health Centre IV	Northeast	Kitgum
9	Kitwe Health Center IV	Health Centre IV	Southwest	Ntungamo
10	Kyabugimbi Health Center IV	Health Centre IV	Southwest	Bushenyi
11	Kabale Regional Referral Hospital	Regional Referral Hospital	Southwest	Kabale
12	Kamuli Health Center IV	Health Centre IV	East Central	Kamuli
