# Supplementary material for: Uganda’s “EID Systems Strengthening” model produces significant gains in testing, linkage, and retention of HIV-exposed and infected infants: An impact evaluation
Source: PLoS One. 2021 Feb 4;16(2):e0246546. doi: 10.1371/journal.pone.0246546 (PMC7861549; doi:10.1371/journal.pone.0246546)
Supplement: S3 File — (PDF) [file pone.0246546.s003.pdf]

S3 File: Data Points for Cohort of HIV-Exposed Infants

No.	Data Point, *by Category* [Fields]	Category Breakdown	Value
**1**	**# HIV-Exposed Infants (HEI) receiving 1st DBS test between June 2011 and May 2014, *total and by PCR result*** [HEI Count, PCR#, PCR result]	HIV-Positive	67
		HIV-Negative	608
		Missing PCR Result	32
		Total	707
**2**	**# Male HEI receiving 1st DBS test, *total and by PCR result*** [HEI Count, Gender, PCR#, PCR result]	HIV-Positive	39
		HIV-Negative	294
		Missing PCR Result	9
		Total	342
**3**	**# Female HEI receiving 1st DBS test, *total and by PCR result*** [HEI Count, Gender, PCR#, PCR result]	HIV-Positive	28
		HIV-Negative	314
		Missing PCR Result	23
		Total	365
**4**	**# HEI receiving 1st DBS test at age of ≤ 2 months, *total and by PCR result*** [HEI Count, Age, Age Bracket, PCR#, PCR Result]	HIV-Positive	17
		HIV-Negative	341
		Missing PCR Result	10
		Total	368
**5**	**# HEI receiving 1st DBS test at age of > 2 months, *total and by PCR result*** [HEI Count, Age, Age Bracket, PCR#, PCR Result]	HIV-Positive	50
		HIV-Negative	265
		Missing PCR Result	17
		Total	332
**6**	**# HEI with no age recorded at 1st DBS test, *total and by PCR result*** [HEI Count, Age, PCR#, PCR Result]	HIV-Positive	0
		HIV-Negative	2
		Missing PCR Result	5
		Total	7

	Data Point, *by Category* [Fields]	Category Breakdown	Value
7	**# Months: Sum total of all ages of HEI at 1st DBS test, *total and HIV+ only*** [HEI, Age Sum, PCR#, PCR Result]	All Tested HEI	2944
		HIV- Positive Only	487
8	**# HEI with documented 'age at 1st DBS test' (months), *total and HIV+ only*** [HEI Count, Age, PCR#, PCR Result]	All Tested HEI	700
		HIV- Positive Only	67
9	**Variance of 'age of HEI at 1st DBS test', *total and HIV+ only*** [HEI Count, Age Distr, PCR#, PCR Result]	All Tested HEI	15.198
		HIV- Positive Only	26.682
	**Data Point, *by Category*** [Fields]	**Category Breakdown**	**Value**
10	**# HEI receiving results of 1st PCR test, *total and by PCR result*** [HEI Count, PCR#, PCR Result, Caregiver Received Results]	Pos	54
		Neg	464
		Result missing	0
		Total	518
11	**# HIV+ infants enrolling in ART clinic, out of those at those testing HIV+ by PCR** [HEI Count, PCR#, PCR Result, Caregiver Received Results, Enrolled at ART Clinic]	Total	50
12	**# HIV+ infants eligible for ART initiation per national guidelines, among HEI testing positive by PCR and enrolling in chronic care at ART clinic** [HEI Count, PCR#, PCR Result, Caregiver Received Results, Enrolled at ART Clinic, Age at ART clinic enrollment, Age at study censorship date, ART eligibility status]	Total	50
13	**# HIV+ infants initiated on ART, among those enrolled at ART clinic and eligible for ART initiation** [HEI Count, PCR#, PCR Result, Caregiver Received Results, Enrolled at ART Clinic, ART eligibility status, ART Initiation, Date of ART initiation, Age at ART initiation, ART Regimen]	Total	46

**14**	**ART Regimens among HIV+ infants started on treatment, *by individual regimen*** [HEI Count, PCR#, PCR Result, Caregiver Received Results, Enrolled at ART Clinic, ART Initiation, ART Regimen, Change in Regimen, Reason for Regimen Change, Transfer Status, Date of Transfer, Location of Transfer]	Tx Regimen AZT-3TC-NVP	40
		Tx Regimen D4T-3TC-EVF	4
		Regimen not documented	2
		Total	46
**15**	**# HIV+ infants active in care at the ART clinic (visiting clinic within the 3 months prior to Nov-2014) among those enrolled in care at ART clinic** [HEI Count, PCR#, PCR Result, Caregiver Received Results, Enrolled at ART Clinic, Date of Enrollment, ART Initiation, ART Regimen, Transfer Status, Date of Transfer, Location of Transfer, Date of most recent visit, Age at most recent visit, Retention status]	Total	44
	**Data Point, *by Category*** [Fields]	**Category Breakdown**	**Value**
**16**	**# Months: Sum total of 'age at ART clinic enrollment' for HIV+ infants enrolled in care** [HEI, PCR#, PCR Result, Caregiver Received Results, Enrolled at ART clinic, Age at ART clinic enrollment (sum)]	Total	407.7
**17**	**# HIV+ infants with documented 'age (months) at enrollment in chronic care at ART clinic'** [HEI Count, PCR#, PCR Result, Caregiver Received Results, Enrolled at ART clinic, Age at ART clinic enrollment]	Total	46
**18**	**Variance of 'age of enrollment at ART clinic' for HIV+ infants** [HEI Count, PCR#, PCR Result, Caregiver Received Results, Enrolled at ART clinic, Age (distr) at ART clinic enrollment]	Total	33.679
	**Data Point, *by Category*** [Fields]	**Category Breakdown**	**Value**
**19**	**# Months: Sum total of 'age at ART initiation' for HIV+ infants started on treatment** [HEI, PCR#, PCR Result, Whether caregiver Received Results, Enrolled at ART Clinic, ART Initiation, Age at ART initiation (Sum)]	Total	412.5

**20**	**# HIV+ infants started on treatment with documented 'age at ART initiation'** [HEI Count, PCR#, PCR Result, Whether caregiver Received Results, Enrolled at ART Clinic, ART Initiation, Age at ART initiation]	Total	42
**21**	**Variance of 'age at ART initiation' for HIV+ infants** [HEI Count, PCR#, PCR Result, Whether caregiver Received Results, Enrolled at ART Clinic, ART Initiation, Age at ART initiation (Distr)]	Total	32.341
	**Data Point, *by Category*** [Fields]	**Category Breakdown**	**Value**
**22**	**# HEI tested, *by feeding status at 1st DBS test*** [HEI Count, PCR#, Age at 1st DBS, Feeding Practice at 1st DBS]	Breastfeeding	655
		Replacement Feeding	44
		Unknown (not documented)	8
		Total	707
**23**	**# HEI breastfeeding at 1st DBS test, *total and by PCR result*** [HEI Count, PCR#, Age at 1st DBS, Feeding Practice at 1st DBS, PCR result]	Pos	56
		Neg	576
		Result missing	23
		Total	655
**24**	**# HEI 'replacement feeding' at 1st DBS test, *total and by PCR result*** [HEI Count, PCR#, Age at 1st DBS, Feeding Practice at 1st DBS, PCR result]	Pos	9
		Neg	31
		Result missing	4
		Total	44
**25**	**# HEI with no documented feeding status at 1st DBS test, *total and by PCR result*** [HEI Count, PCR#, Age at 1st DBS, Feeding Practice at 1st DBS, PCR result]	Pos	2
		Neg	1
		Result missing	5
		Total	8
**26**	**# HEI receiving 1st PCR results among those breastfeeding at DBS and testing negative, *total and by age bracket (< 13 mo at DBS test ≥)*** [HEI Count, PCR#, PCR Result, Age at 1st DBS, Age Bracket, Feeding Practice at 1st DBS]	Neg and < 13 mo at DBS	352
		Neg and ≥ 13 mo at DBS	90
		Total	442

**27**	**# HEI with negative 1st PCR who received 2nd confirmatory PCR after cessation of breastfeeding, *total and by 2nd-PCR result*** [HEI Count, PCR#, PCR Result, Age at 1st DBS, Age Bracket, Feeding Practice at 1st DBS, Date stopped breastfeeding, Age stopped breastfeeding, Due date for 2nd DBS draw, Whether 2nd DBS received, Age at 2nd DBS, 2nd PCR result, Whether caregiver received 2nd PCR result]	HIV-negative (2nd PCR)	168
		HIV-positive (2nd PCR)	23
		Result missing (2nd PCR)	1
		Total (2nd PCR)	192
**28**	**# HEI receiving results of 2nd confirmatory PCR after cessation of breastfeeding, *total and by 2nd-PCR result*** [HEI Count, PCR#, PCR Result, Age at 1st DBS, Age Bracket, Feeding Practice at 1st DBS, Due date for 2nd DBS draw, Received 2nd DBS, Age at 2nd DBS, 2nd PCR result, Whether caregiver received 2nd PCR result]	HIV-negative (2nd PCR)	115
		HIV-positive (2nd PCR)	18
		Result missing (2nd PCR)	0
		Total (2nd PCR)	133
	**Data Point, *by Category*** [Fields]	**Category Breakdown**	**Value**
**29**	**# of HIV+ infants diagnosed through 2nd PCR post-breastfeeding, who were enrolled in ART clinic** [HEI Count, PCR#, PCR Result, Feeding Practice at 1st DBS, Received 2nd DBS, 2nd PCR result, Whether caregiver received 2nd PCR result, Enrolled at ART clinic]	Total (2nd PCR HIV+)	18
**30**	**# of HIV+ infants diagnosed through 2nd PCR after cessation of breastfeeding, who were initiated on ART** [HEI Count, PCR#, PCR Result, Feeding Practice at 1st DBS, Received 2nd DBS, 2nd PCR result, Whether caregiver received 2nd PCR result, Enrolled at ART clinic, ART Initiation, Age at ART initiation]	Total (2nd PCR HIV+)	16
**31**	**# of HIV+ infants diagnosed through 2nd PCR post-breastfeeding, who were active in care (made visit within the 3 months prior to 10/31/2014)** [HEI Count, PCR#, PCR Result, Feeding Practice at 1st DBS, Received 2nd DBS, 2nd PCR result, Whether caregiver received 2nd PCR result, Enrolled at ART clinic, Age at ART clinic enrollment, ART Initiation, ART Regimen, Transfer Status, Date of Transfer, Location of Transfer, Date of most recent visit, Age at most recent visit, Retention status]	Total (2nd PCR HIV+)	17

	Data Point, *by Category* [Fields]	Category Breakdown	Value
32	**Total # of DBS tests (1st and 2nd PCR) done for HEI in the cohort during review period** [HEI Count, PCR#, PCR Result, 2nd PCR result]	Total	899
	**Data Point, *by Category*** [Fields]	**Category Breakdown**	**Value**
33	**# of HIV-positive pregnant women attending ANC** [HIV+ pregnant women count, date of 1st ANC visit, gestational age at 1st ANC visit, expected delivery date]	Total	334
34	**# of HIV+ pregnant women attending ANC whose infants received DBS test after birth** [HIV+ pregnant women count, date of 1st ANC visit, gestational age at 1st ANC visit, expected delivery date, date of delivery, location of delivery, expected age of infant at 6 weeks old, whether infant received DBS test, PCR result, PCR#]	Total	175
	**Data Point, by Category** [Fields]	**Category Breakdown**	**Value**
35	**# Days: Sum total of all "days between DBS sample draw and sample dispatch to reference lab" for tested HEI (1st and 2nd PCR)** [HEI, PCR#, PCR Result, Date of sample collection, Date of sample dispatch, Turnaround time to dispatch (Sum)]	Total	3633
36	**# HEI with data documented for "days between DBS sample draw and sample dispatch to reference lab" for tested HEI (1st and 2nd PCRs)** [HEI count, PCR#, PCR Result, Date of sample collection, Date of sample dispatch]	Total	738
37	**Variance of 'days between DBS sample draw and sample dispatch to reference lab" (1st and 2nd PCRs)** [HEI, PCR#, PCR Result, Date of sample collection, date of sample dispatch, Turnaround time to dispatch (Distr)]	Total	16.245
	**Data Point, *by Category*** [Fields]	**Category Breakdown**	**Value**
38	**# Days: Sum total of all "days between DBS sample draw and return of results to facility" for tested HEI (1st and 2nd PCR)** [HEI, PCR#, PCR Result, Date of sample collection, Date of sample dispatch, Date of sample return to facility, Turnaround time from DBS to result return (Sum)]	Total	19822

**39**	**# HEI with data documented for "days between DBS sample draw and return of results to facility" for tested HEI (1st and 2nd PCRs)** [HEI count, PCR#, PCR Result, Date of sample collection, Date of sample return to facility, Turnaround time from DBS to result return]	Total	791
**40**	**Variance of "number of days between DBS and return of results to health facility" for tested HEI (1st and 2nd PCRs)** [HEI, PCR#, PCR Result, Date of sample collection, Date of sample dispatch, Date of sample return to facility, Turnaround time from DBS to result return (Distr)]	Total	240.215
	**Data Point, *by Category*** [Fields]	**Category Breakdown**	**Value**
**41**	**# Days: Sum total "days between DBS test and provision of results to caregiver" for tested HEI who received results (1st and 2nd PCRs)** [HEI, PCR#, PCR Result, Date of sample collection, Date of sample dispatch, Whether Caregiver Received Results, Date Caregiver Received Results, Turnaround time from DBS to caregiver receipt of results (Sum)]	Total	24310
**42**	**# HEI with data documented for "number of days between DBS and provision of results to caregiver" for tested HEI who received results (1st and 2nd PCR)** [HEI count, PCR#, PCR Result, Date of sample collection, Whether Caregiver Received Results, Date Caregiver Received Results, Turnaround time from DBS to caregiver receipt of results]	Total	568
**43**	**Variance of "number of days between DBS and provision of results to caregiver" for tested HEI who received results (1st and 2nd PCR)** [HEI, PCR#, PCR Result, Date of sample collection, Whether Caregiver Received Results, Date Caregiver Received Results, Turnaround time from DBS to caregiver receipt of results (Distr)]	Total	657.604
	**Data Point, *by Category*** [Fields]	**Category Breakdown**	**Value**
**44**	**# Visits: Sum total of all "clinic visits made before retrieving results" for HEI who received 1st PCR results** [HEI, PCR#, PCR Result, Whether Caregiver Received Results, Date Caregiver Received Results, # visits to receive results (Sum)]	Total	559
**45**	**# HEI receiving 1st PCR results with data documented for "clinic visits made before retrieving results"** [HEI count, PCR#, PCR Result, Whether Caregiver Received Results, Date Caregiver Received Results, # visits to receive results]	Total	467

**46**	**Variance of "clinic visits made before retrieving results" for HEI who received 1st PCR results** [HEI, PCR#, PCR Result, Whether Caregiver Received Results, Date Caregiver Received Results, # visits to receive results (Distr)]	Total	0.282
